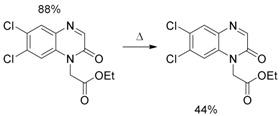# Carbene Transfer Reactions Catalysed by Dyes of the Metalloporphyrin Group

**DOI:** 10.3390/molecules23040792

**Published:** 2018-03-29

**Authors:** Mário M. Q. Simões, Daniel T. G. Gonzaga, Mariana F. C. Cardoso, Luana da S. M. Forezi, Ana T. P. C. Gomes, Fernando de C. da Silva, Vítor F. Ferreira, Maria G. P. M. S. Neves, José A. S. Cavaleiro

**Affiliations:** 1 Department of Chemistry & Organic Chemistry, Natural Products and Food Stuffs Unit (QOPNA), University of Aveiro, 3810-193 Aveiro, Portugal; ana.peixoto@ua.pt (A.T.P.C.G.); gneves@ua.pt (M.G.P.M.S.N.); 2 Departamento de Química Orgânica, Universidade Federal Fluminense, Niterói 24020-150, RJ, Brazil; danieltadeugonzaga@yahoo.com.br (D.T.G.G.); marianafcc83@hotmail.com (M.F.C.C.); luanaforezi@hotmail.com (L.d.S.M.F.); gqofernando@vm.uff.br (F.d.C.d.S.); 3 Departamento de Tecnologia Farmacêutica Química, Universidade Federal Fluminense, Niterói 24241-002, RJ, Brazil

**Keywords:** carbenes, porphyrins, metalloporphyrins, cyclopropanation, cyclopropenation, olefination, X-H insertion

## Abstract

Carbene transfer reactions are very important transformations in organic synthesis, allowing the generation of structurally challenging products by catalysed cyclopropanation, cyclopropenation, carbene C-H, N-H, O-H, S-H, and Si-H insertion, and olefination of carbonyl compounds. In particular, chiral and achiral metalloporphyrins have been successfully explored as biomimetic catalysts for these carbene transfer reactions under both homogeneous and heterogeneous conditions. In this work the use of synthetic metalloporphyrins (MPorph, M = Fe, Ru, Os, Co, Rh, Ir, Sn) as homogeneous or heterogeneous catalysts for carbene transfer reactions in the last years is reviewed, almost exclusively focused on the literature since the year 2010, except when reference to older publications was deemed to be crucial.

## 1. Introduction

Carbenes are electrically neutral species formulated as RR’C: in which the carbon is covalently bonded to two univalent groups or to a divalent group, and bears two nonbonding electrons. Methylene (H_2_C:) is the simplest carbene and the two nonbonding electrons may be spin-paired (singlet state) or spin-non-paired (triplet state) [1]. Carbene transfer reactions are very important transformations in organic synthesis, allowing one to generate cyclopropane derivatives and other structurally challenging products. It should be stressed out that no native metalloenzymes for accomplishing that task have been revealed yet, but by exploiting substrate promiscuity and protein engineering, structurally manipulated heme-containing cytochrome P450 enzymes were described to catalyse carbene transfer reactions in their non-native chemical form [2,3,4,5,6,7,8,9,10,11,12,13]. Moreover, Hartwig and co-workers [14] have prepared modified myoglobins containing several abiological metals, the most active being an Ir(Me) site that catalyses the functionalization of C-H bonds to form C-C bonds by carbene insertion and adding carbenes to β-substituted vinylarenes and unactivated aliphatic α-olefins. On the other hand, Fasan and co-workers [15,16,17,18,19,20,21,22] recently demonstrated the ability of engineered myoglobin variants to efficiently catalyse carbene N-H, S-H, and C-H insertions, cyclopropanation, and olefination of aldehydes as examples of efficient and selective biocatalytic strategies. Meanwhile, several other promising biocatalytic approaches for carbene insertion reactions have been published [23,24,25,26,27]. Similar catalytic activity was observed, in the last decades, by using several synthetic metalloporphyrins (MPorph, M = Fe, Ru, Os, Co, Rh, Ir, Sn), including chiral or supported porphyrin metal complexes, as catalysts for carbene transfer reactions [28,29,30,31,32,33,34,35,36,37,38]. Taking into account the high significance of the mentioned carbene synthetic methodologies this review is mainly focused on carbenes’ developments in the last years.

## 2. Cyclopropanation Reactions

**Scheme 1:**
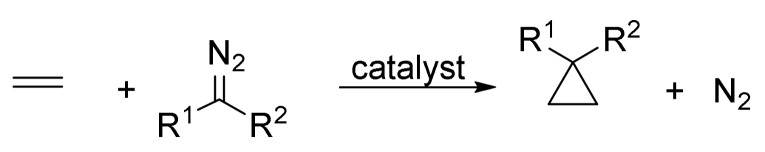
The catalysed cyclopropanation reaction as an addition of a carbene group, from a diazo compound, to any alkene.

Cyclopropane derivatives are useful building blocks in organic synthesis, presenting several potential applications in organic chemistry. Amongst the well-known methods to prepare cyclopropane derivatives, the addition of a carbene group from a diazo compound to an alkene (Scheme 1) has been deeply studied in the last decades [28,30,39,40,41,42,43,44,45]. Thus, the one-pot reaction of diazo compounds with alkenes represents a valuable strategy to prepare cyclopropanes, important building blocks both for the synthesis of organic compounds and also due to their intrinsic pharmaceutical properties [32].

**Scheme 2:**
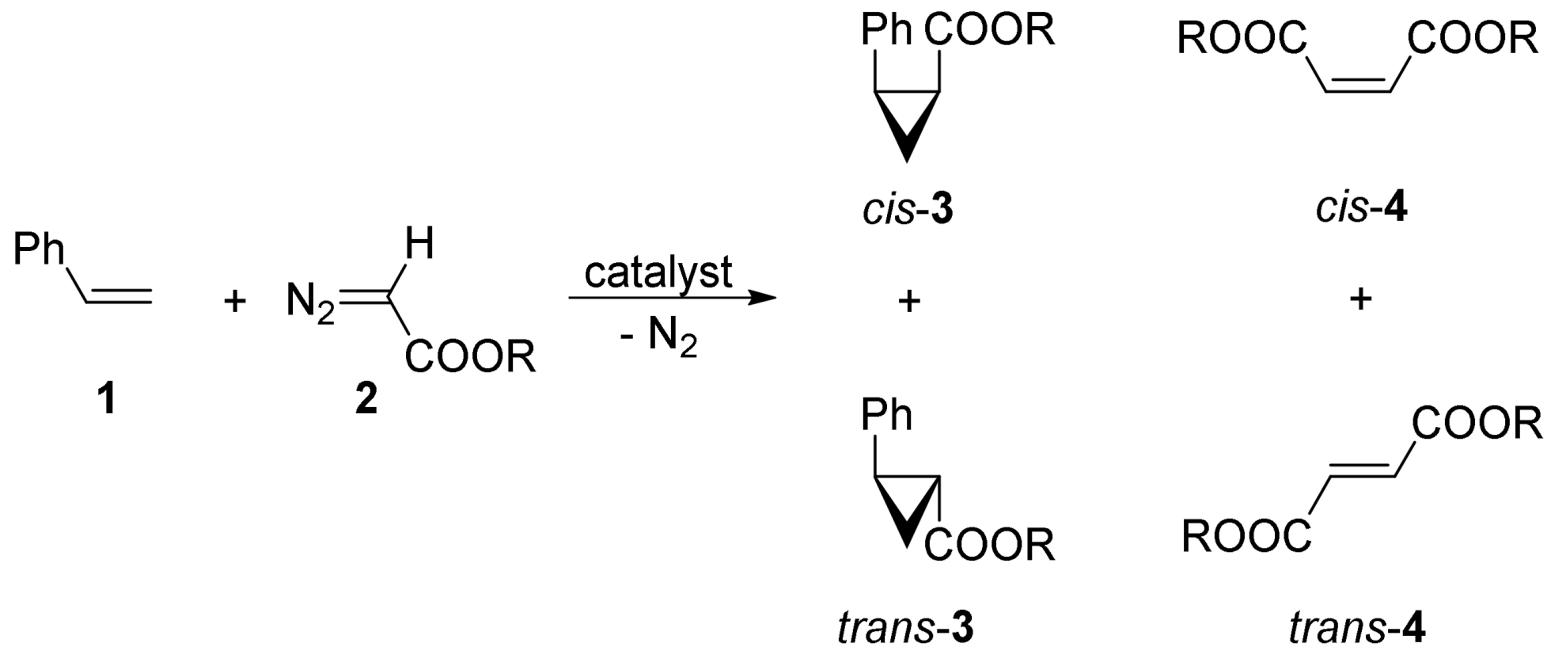
The cyclopropanation of styrene (**1**) with methyl diazoacetate (MDA) or ethyl diazoacetate (EDA) (**2**, R = Me or Et) as a benchmark reaction.

The cyclopropanation of styrene (**1**) with methyl diazoacetate (MDA) or ethyl diazoacetate (EDA) (**2**, R = Me or Et) serves as the benchmark reaction for the evaluation of almost any new catalyst (Scheme 2). Typically, a diastereoisomeric mixture of cyclopropanes *cis*-**3** and *trans*-**3** is obtained (each existing as a pair of enantiomers), accompanied by the undesired carbene dimers *cis*-**4** and *trans*-**4**. The relative amounts of the later products increase when the cyclopropanation becomes a slow transformation [46].

**Scheme 3:**
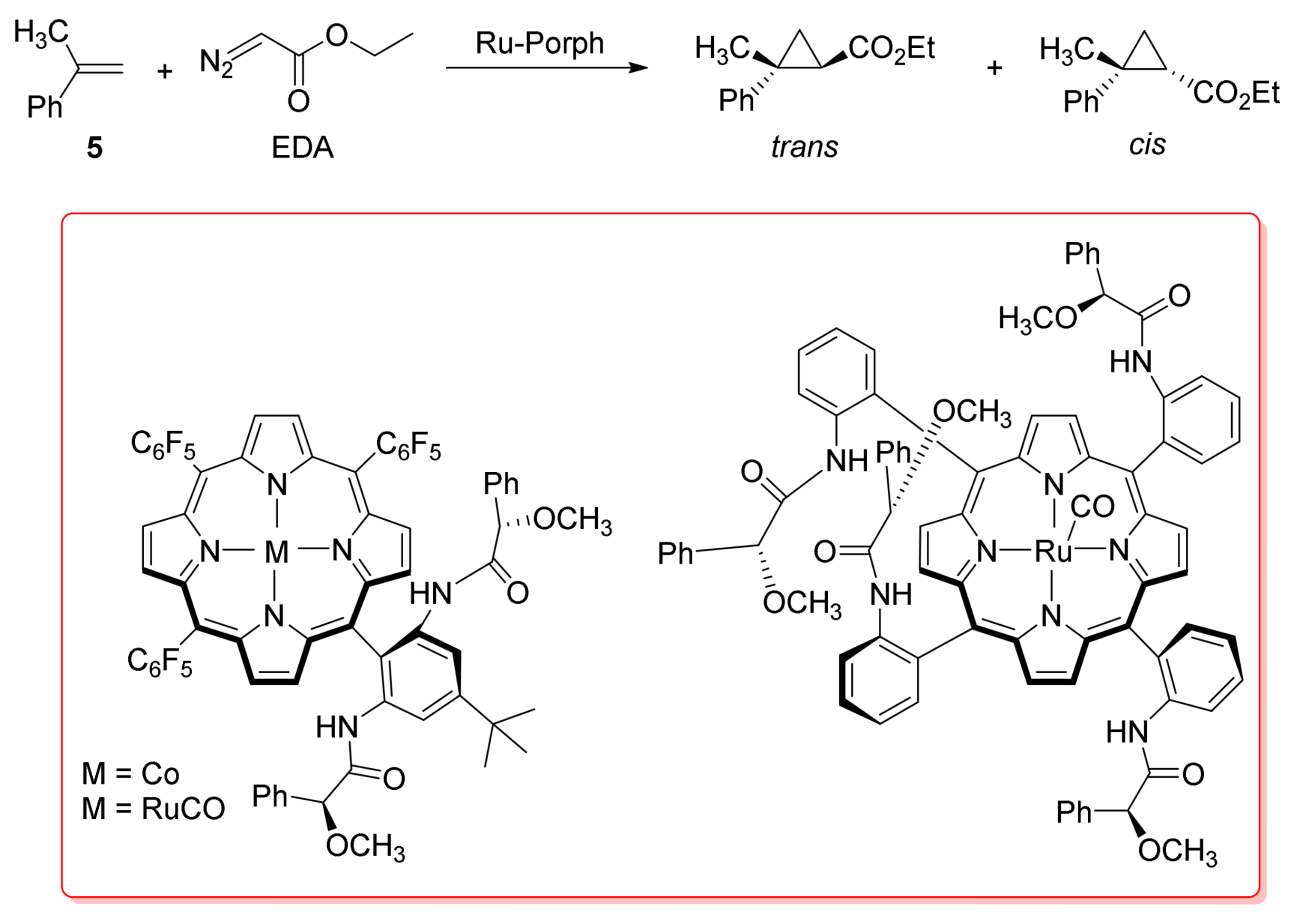
Intermolecular cyclopropanation of α-methylstyrene (**5**) by EDA catalysed by the Ru complexes of both the diamido and tetraamidoporphyrins [47].

In recent years, Gallo and co-workers have been particularly focused on the study of carbene transfer reactions catalysed by metalloporphyrins, with emphasis on the active role of the porphyrin macrocycle in the stereocontrol of the reactions, concerning both diastereoselectivity and enantioselectivity [29,32]. Some years ago, this research group reported the preparation of cobalt and ruthenium chiral amidoporphyrin derivatives. The catalytic properties of those products were then evaluated in cyclopropanation transformations. The cobalt diamidoporphyrin complex did not lead to the formation of cyclopropane products; however, with the ruthenium complexes (Scheme 3) the expected products from the reaction of α-methylstyrene (**5**) by EDA were obtained as diastereoisomeric mixtures in 91% to 95% yields. Transformations took place with poor diastereo- and enantioselectivities. When using the Ru-diamidoporphyrin complex a (66/34%) diastereoisomeric *cis/trans* mixture was obtained while with the Ru-tetraamidoporphyrin an inversion in the ratio *cis/trans* value (34/66%) was obtained [47].

**Figure 1:**
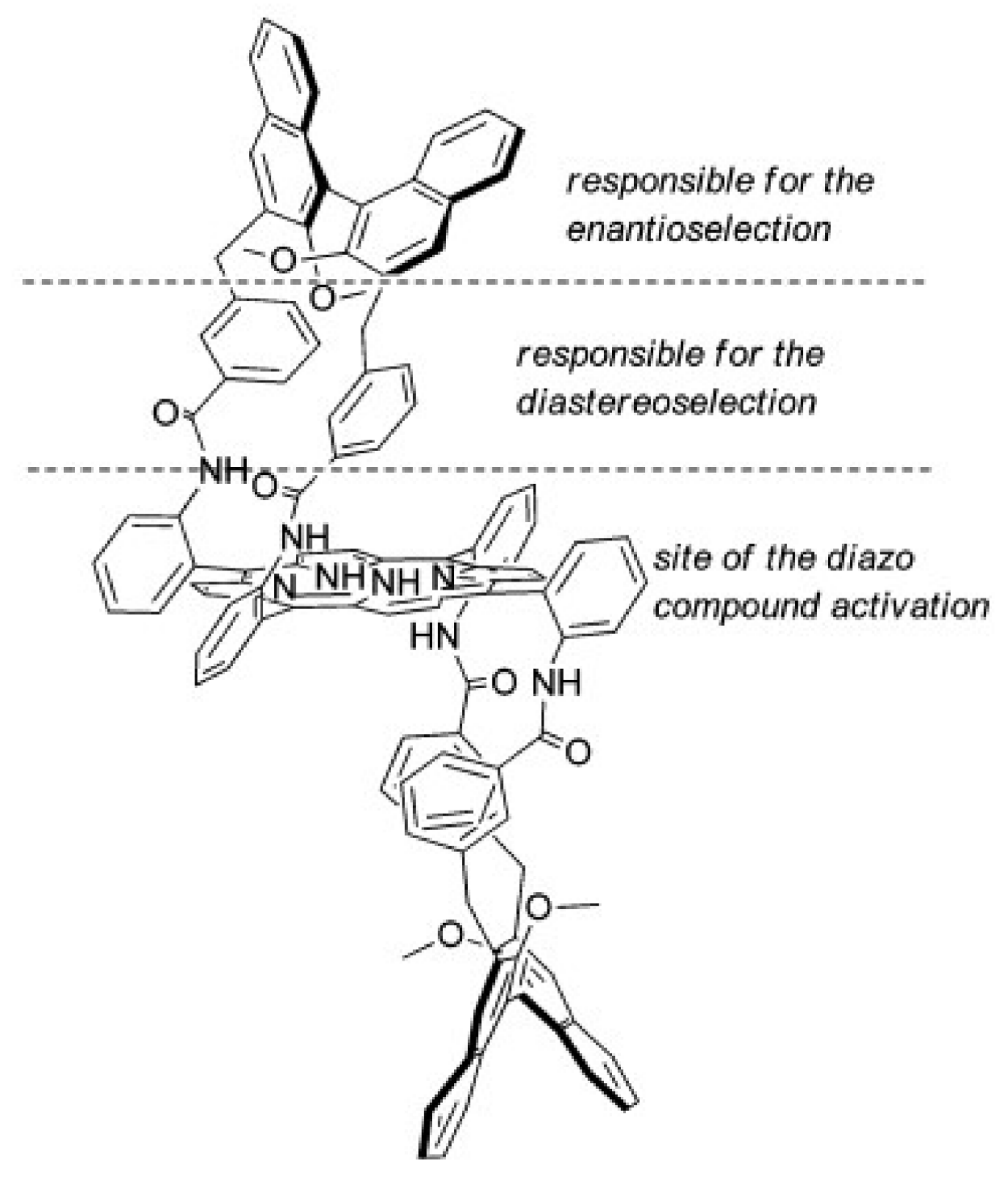
The totem-like structure of the free base chiral porphyrin reported by Gallo and co-workers. Reproduced from [50] with permission from the Centre National de la Recherche Scientifique (CNRS) and The Royal Society of Chemistry.

Most recently, Gallo and co-workers described a new chiral iron(III) porphyrin catalyst capable of performing the stereoselective cyclopropanation of α-methylstyrene (**5**) with excellent yields (up to 99%), enantio- and diastereoselectivities (ee*_trans_* up to 87%, *trans/cis* ratios up to 99:1) and outstanding TON and TOF values (up to 20,000 and 120,000 h^−1^ respectively) [48]. Moreover, further studies were carried out to evaluate the reaction scope by testing the reactivity of several diazo derivatives of the general formula (RO_2_C)CH=N_2_ (R = Et; *i*Pr; Pr; *t*Bu) towards differently substituted alkenes. The study of the cyclopropanation reaction revealed that the porphyrin skeleton may be compared to a totem-like structure (Figure 1), whose parts are independently responsible for the observed enantio- and diastereoselectivities.

The iron centre of the tetrapyrrolic macrocycle activates the diazo compound; its C_2_-symmetrical feature induces high *trans*-diastereoisomeric ratios, whereas the chiral hat controls the enantiocontrol leading to a diastereoselectivity enhancement (Figure 1) [49,50]. The aforementioned results led the authors to prepare other C_2_-symmetrical chiral iron(III) porphyrins, where the chiral binaphthyl unit was replaced by an aminoacid moiety. The results obtained confirmed that the diastereoselectivity was mainly ruled by the achiral structural parts of the porphyrin which originate a 3D arrangement to the whole ligand. The presence of aminoacid residues in the chiral hat did not significantly contribute for selecting the enantiomeric path, since modest enantioselectivities were attained [50].

**Scheme 4:**
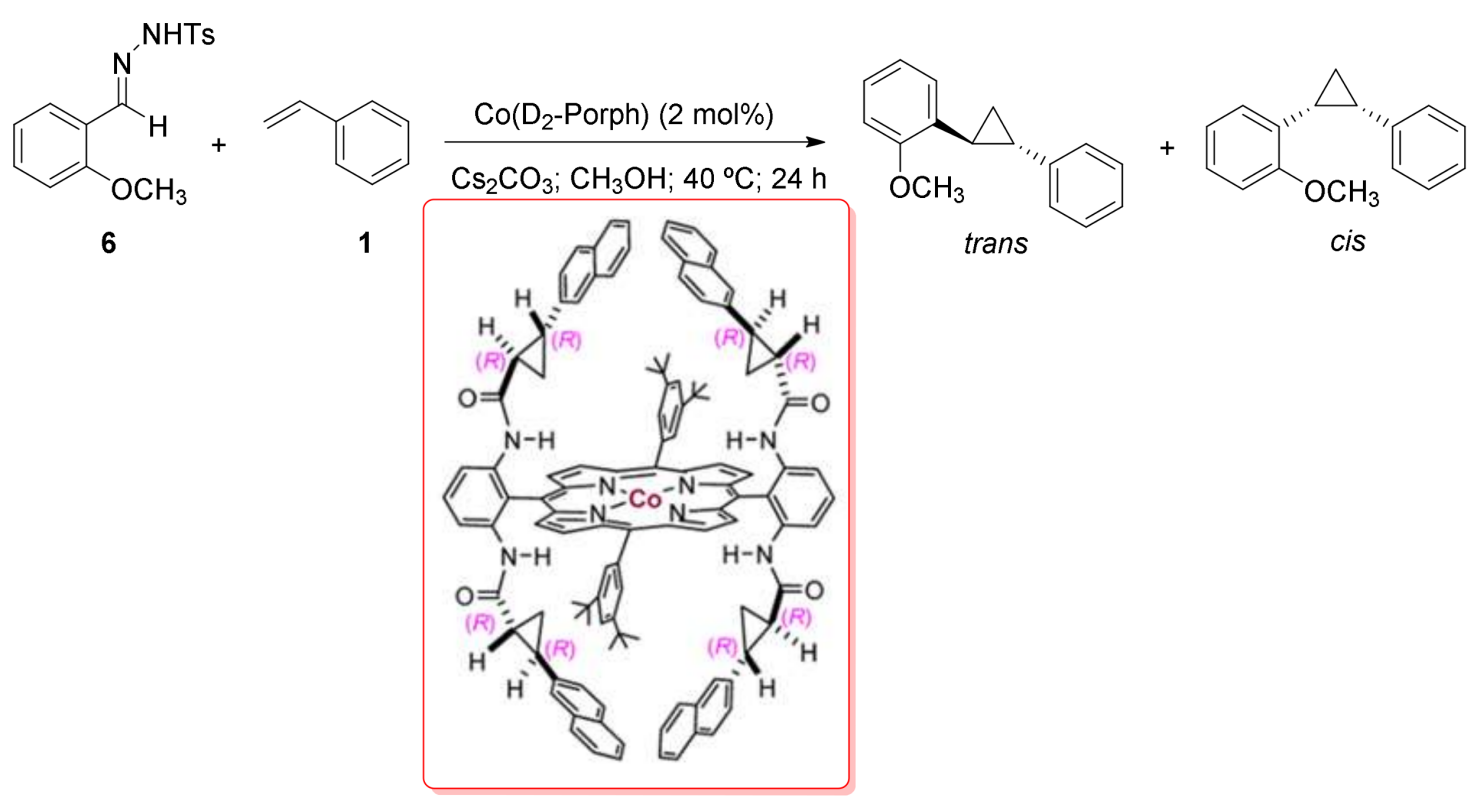
The chiral naphthyl-substituted tetraamidoporphyrin responsible for the higher enantioselectivity (99% *ee*) obtained in styrene (**1**) cyclopropanation with 2-methoxybenzaldehyde tosylhydrazone (**6**) at 83% yield and 95:5 *trans/cis* ratio. Adapted with permission from [51]. Copyright (2017) American Chemical Society.

Several Co(II) complexes of D_2_-symmetric chiral tetraamidoporphyrins were disclosed by Zhang and co-workers as efficient catalysts able to activate several *N*-arylsulfonyl hydrazones for the asymmetric intermolecular cyclopropanation of a wide range of alkenes, affording the corresponding cyclopropanes in high yields, sometimes with effective control of both diastereo- and enantioselectivity. The higher enantioselectivity (99% *ee*) was obtained for styrene (**1**) cyclopropanation with the 2-methoxybenzaldehyde tosylhydrazone (**6**) at 83% yield and 95:5 *trans/cis* ratio (Scheme 4). According to the authors, the Co(II) metalloradical intermediate represents the first catalytic species capable of effectively utilize donor-substituted diazo reagents, generated in situ from *N*-arylsulfonyl hydrazones in the presence of base, for asymmetric alkene cyclopropanation [51].

**Scheme 5:**
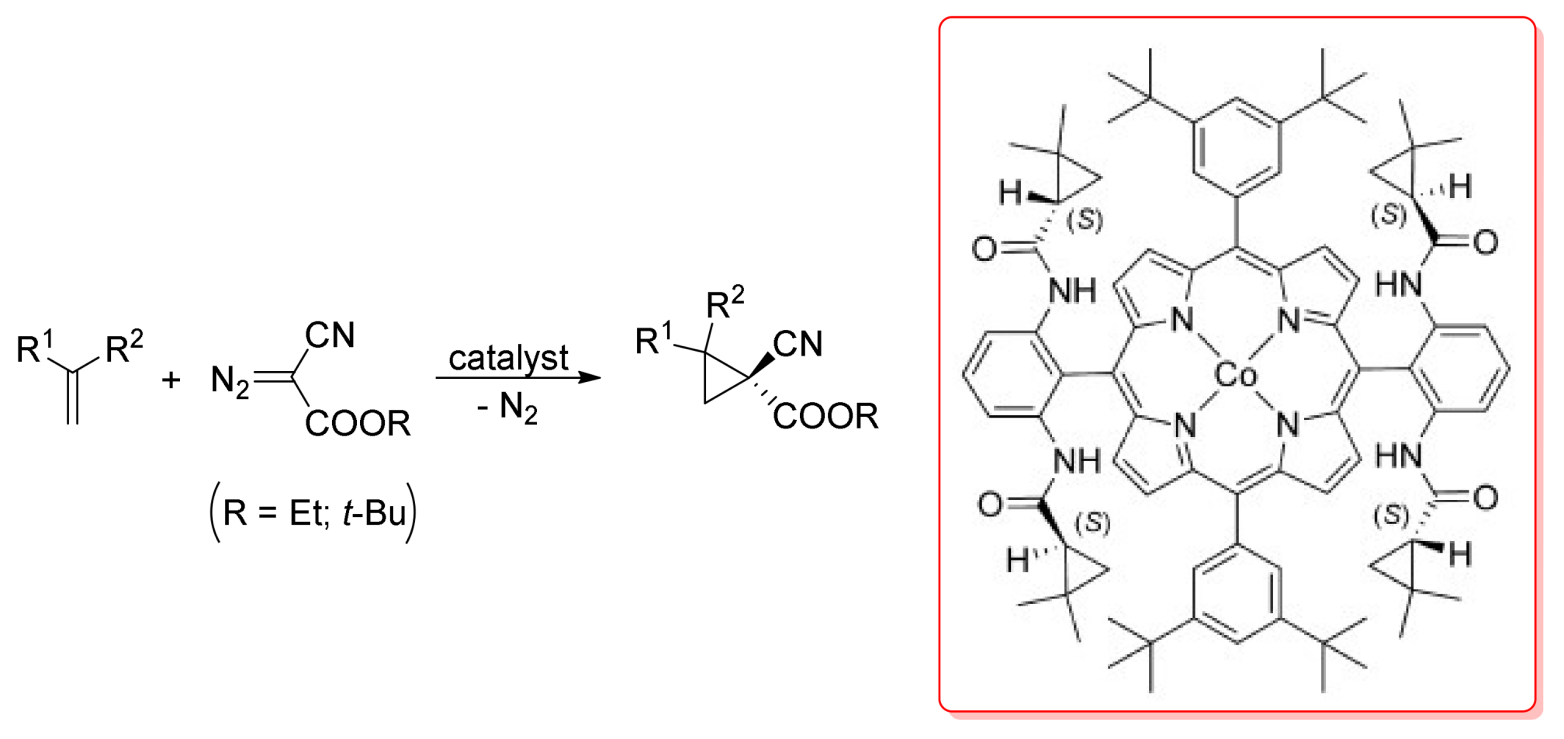
Asymmetric cyclopropanation of olefins with α-cyanodiazoacetates. Adapted with permission from [53]. Copyright (2010) American Chemical Society.

Earlier, the same group had already referred the efficiency of Co(II) complexes of D_2_-symmetric chiral amidoporphyrins in the catalytic asymmetric intermolecular cyclopropanation of both aromatic and aliphatic substituted olefins possessing different electronic profiles with α-cyanodiazoacetates (Scheme 5). The cyclopropanation of styrene at 25 °C in hexane with ethyl α-cyanodiazoacetate (ECDA) afforded the corresponding cyclopropane derivative in 99% yield with a *trans/cis* ratio of 88:12 and 74% *ee*. In analogous reaction conditions, when using *tert*-butyl α-cyanodiazoacetate (*t*-BCDA) instead of ECDA, it was obtained the single *trans*-cyclopropane derivative in 91% *ee*, in a lower yield (89%). However carrying out the reaction at 0 °C the enantioselectivity had a slight improvement to 95% *ee*. Surprisingly, even at lower temperature (−20 °C), the reaction took place with 96% yield and an increase on the enantiocontrol, with 98% *ee* and full *trans*-diastereoselectivity [52].

**Scheme 6:**
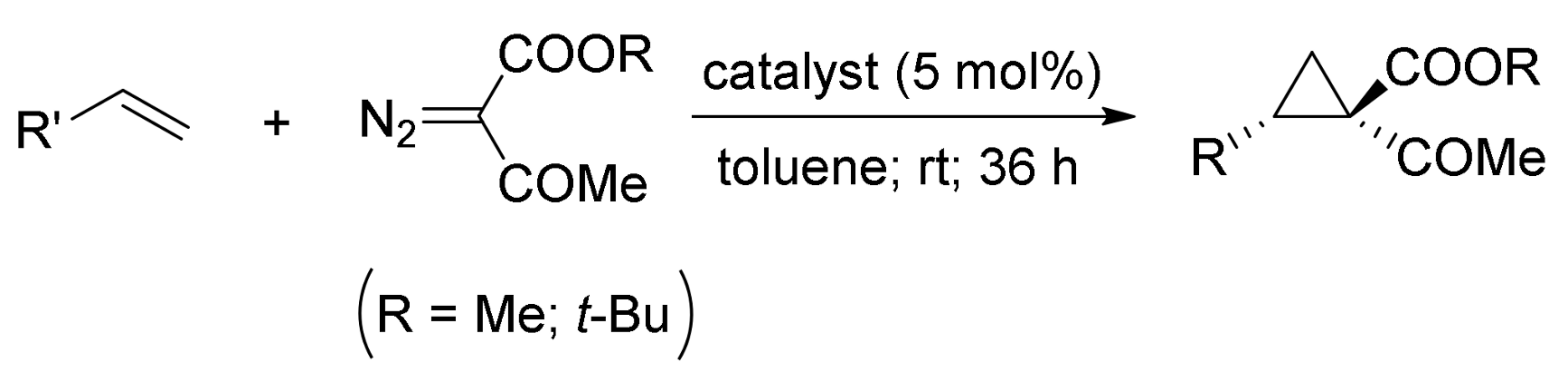
Asymmetric cyclopropanation of olefins with α-ketodiazoacetates.

The same Co(II) complex of the D_2_-symmetric chiral amidoporphyrin present on Scheme 5 showed to be highly effective for asymmetric olefin cyclopropanation with α-ketodiazoacetates (KDA), acceptor/acceptor-substituted diazo reagents bearing two α-carbonyl groups, thus resulting on the production of chiral 1,1-cyclopropaneketoesters bearing both ketone and ester functionalities at the geminal position (Scheme 6). The asymmetric cyclopropanation of styrene with methyl acetodiazoacetate (MADA) could be achieved under mild reaction conditions (at ambient temperature with the alkene as the limiting reagent without slow addition of the diazo compound), thus affording the desired cyclopropane in 99% yield with *E/Z* ratio of 79:21 and 63% *ee*. By employing the bulkier *tert*-butyl acetodiazoacetate (*t*-BADA) reagent the authors were able to significantly improve the diastereoselectivity (*E/Z* ratio of 96:4, 92% *de*) of the catalytic system while at the same time increasing its enantioselectivity (94% *ee* for 1*R*,2*S*), although in a lower yield (88%).

The latter asymmetric catalytic system might be used in the cyclopropanation of a variety of olefins. For instance, and despite the position and electronic properties of the substituents (electron-donating groups such as CH_3_ or OCH_3_, as well as electron-withdrawing Br, CF_3_, or NO_2_ groups), styrene derivatives might be cyclopropanated with *t*-BADA forming the expected cyclopropaneketoesters in high yields, diastereoselectivity and enantioselectivity [54].

**Figure 2:**
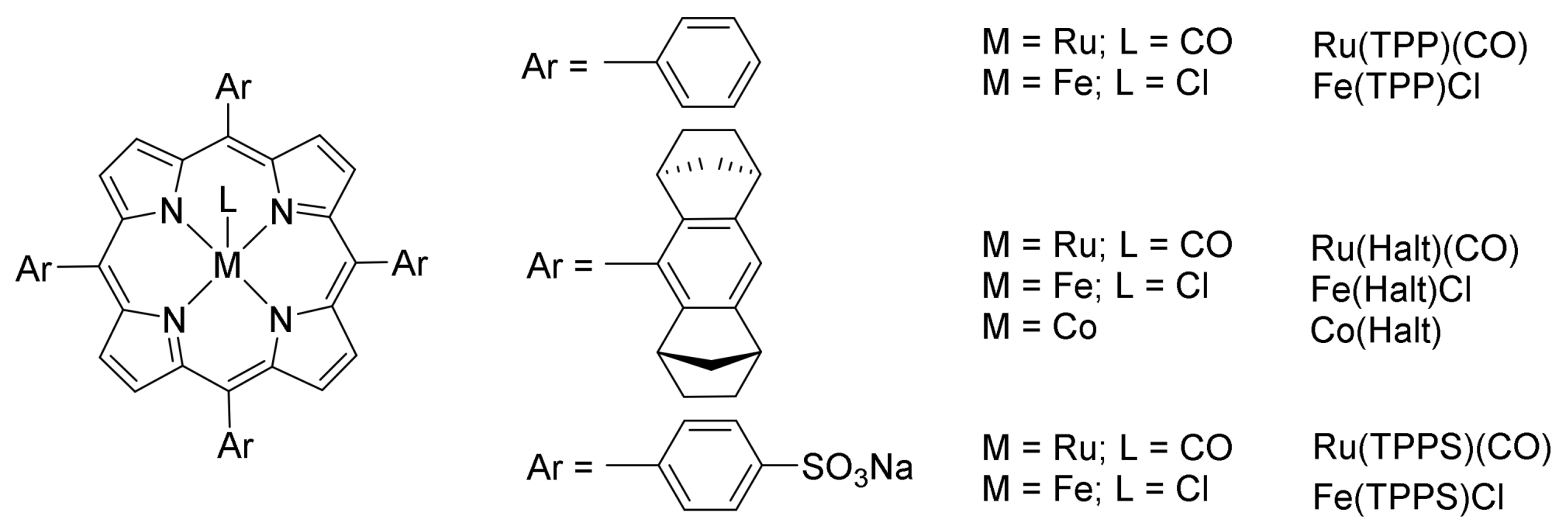
Iron, ruthenium, and cobalt porphyrin complexes.

The Co(II) complex of the so-called Halterman porphyrin [5,10,15,20-tetrakis[(1*S*,4*R*,5*R*,8*S*)-1,2,3,4,5,6,7,8-octahydro-1,4:5,8-dimethanoanthracene-9-yl]porphyrinatocobalt(II)] (Figure 2, Co(Halt)) was used as catalyst in the cyclopropanation of styrene with ethyl diazoacetate. Up to 80:20 *trans:cis* diastereoisomeric ratio and 82% *ee* were achieved with 96% total yield within 1 h at ambient temperature, by using this Co(II) chiral porphyrin complex as catalyst. On the other hand, the cyclopropanation of 1,1-diphenylethylene with EDA catalysed by the same Co(II) porphyrin gave the corresponding cyclopropane in moderate yield (62%) and 50% *ee* [55].

**Scheme 7:**
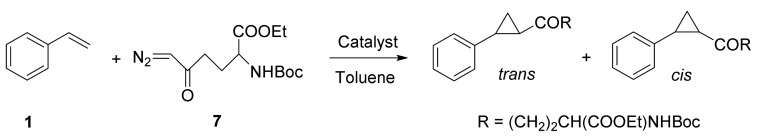
The intermolecular cyclopropanation of styrene (**1**) with the *N*- and *O*-protected 6-diazo-5-oxo-l-norleucine (DON) (**7**) at ambient temperature in toluene and in the presence of metalloporphyrins.

Simonneaux and co-workers described the catalysed carbene transfer from the *N*- and *O*-protected 6-diazo-5-oxo-l-norleucine (DON) (**7**) in the presence of iron and ruthenium porphyrin complexes, giving rise to intermolecular reactions such as cyclopropanation or N-H and S-H insertion reactions. The cyclopropanation of styrene (**1**) with the *N*- and *O*-protected DON at ambient temperature in toluene and in the presence of the Ru(TPP)(CO) (Figure 2) catalyst afforded the corresponding cyclopropane product (Scheme 7) with 72% yield and high diastereoselectivity (*trans/cis* = 99/1) after 5 h, with the simultaneous formation of the dimer (21%) resulting from coupling of two carbene precursors. An increased yield (85%), although with lower selectivity (*trans/cis* = 91/9), was observed when using Fe(TPP)Cl instead of Ru(TPP)(CO) as catalyst for the same reaction time. With catalysts Ru(TPP)(CO) and Fe(TPP)Cl no enantiomeric excess was observed [56].

The authors also studied the asymmetric cyclopropanation reaction catalysed by the Halterman chiral porphyrins, identified here as Fe(Halt)Cl and Ru(Halt)(CO) (Figure 2). The cyclopropane product yield was higher with the iron chiral porphyrin (95%) relatively to the ruthenium chiral porphyrin (75%) and the enantioselectivity was quite good for both chiral catalysts (80% *ee*).

**Figure 3:**
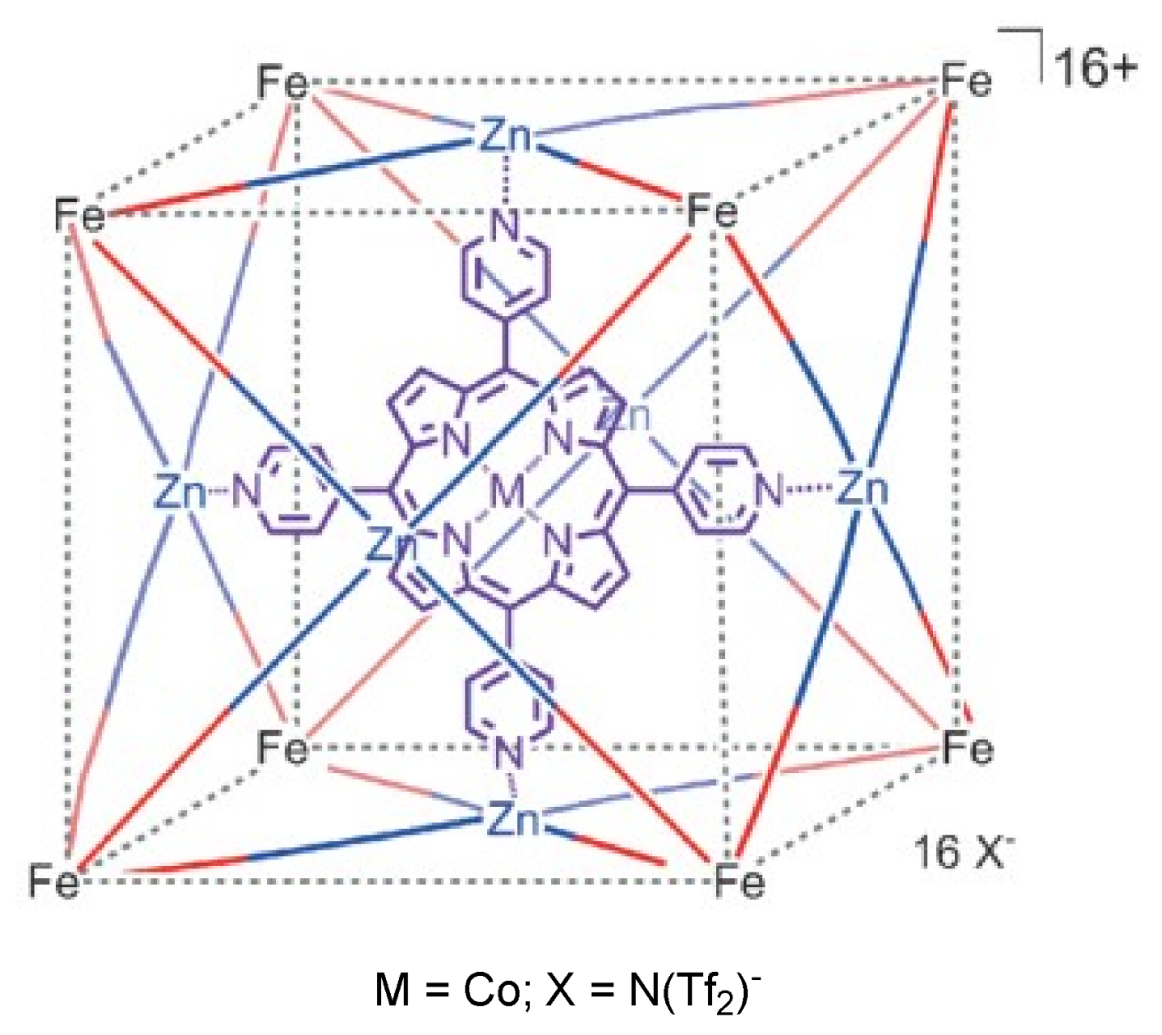
Cobalt porphyrin catalyst encapsulated in a cubic M_8_L_6_ cage. Adapted from [58] with permission from John Wiley and Sons.

Bruin and co-workers have prepared a cobalt porphyrin catalyst encapsulated in a cubic M_8_L_6_ cage by using the so-called “molecular ship-in-a-bottle” approach. The soluble M_8_L_6_ encapsulated catalyst (Figure 3) allows cyclopropanation reactions in aqueous media (acetone:water 1:5) and revealing size-selectivity for cyclopropanation of styrene derivatives with alkyl diazoacetates, although in modest yields and *trans/cis* diastereoisomeric ratios. Bulky substrates reacted slower than non-bulky ones, thus allowing size-selective competition reactions [57,58].

**Figure 4:**
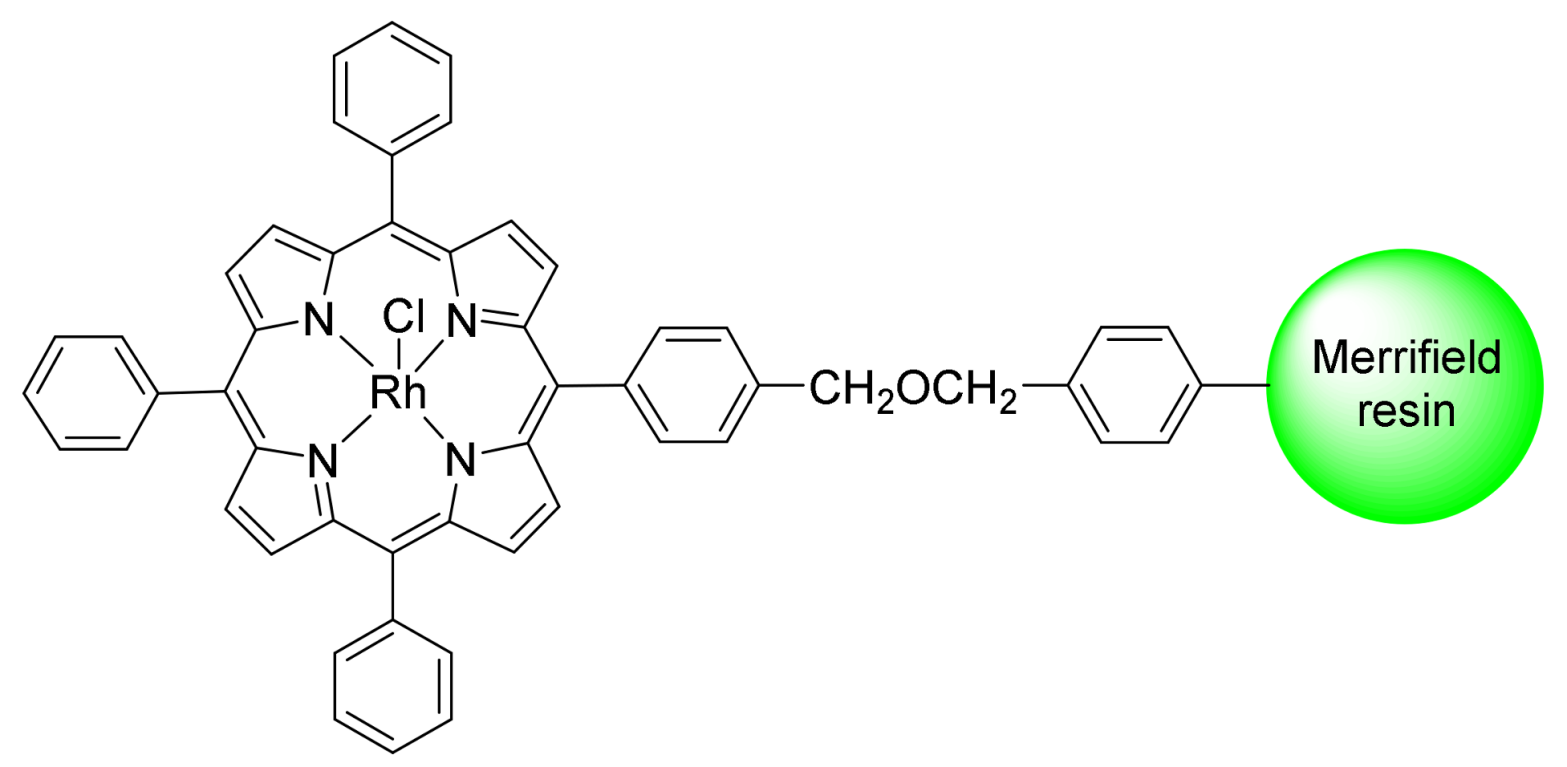
The rhodium porphyrin was bound to the support by using the Williamson method for the synthesis of ethers, affording a loading around 1 mmol per gram of solid.

Tagliatesta and co-workers reported a heterogeneous system based on a *meso*-tetraphenylporphyrin rhodium(III) chloride, bound to a Merrifield resin (Figure 4), to catalyse the cyclopropanation reaction of several olefins, giving good yields for the final products. The linkage between the Rh(III) porphyrin and the solid support allowed the separation of the functionalized resin from the reaction mixtures by vacuum filtration and further reuse, although with significant loss of activity in the second cycle [59].

Having in mind the enzymes’ action, the potential catalytic action played by DNA-derivatives, such as the possibility of having a transition metal ion complex surrounded by a DNA framework, which would furnish another chiral coordination sphere, seems to be a promising approach to the design of highly active and selective catalysts. Based on previous DNA-based catalysis works, and on their own experience [60], Roelfes and co-workers recently introduced a novel hybrid catalyst, based on the interaction between DNA and a cationic iron(III) porphyrin. In that way interesting catalytic enantioselective cyclopropanation reactions were observed [61].

**Scheme 8:**
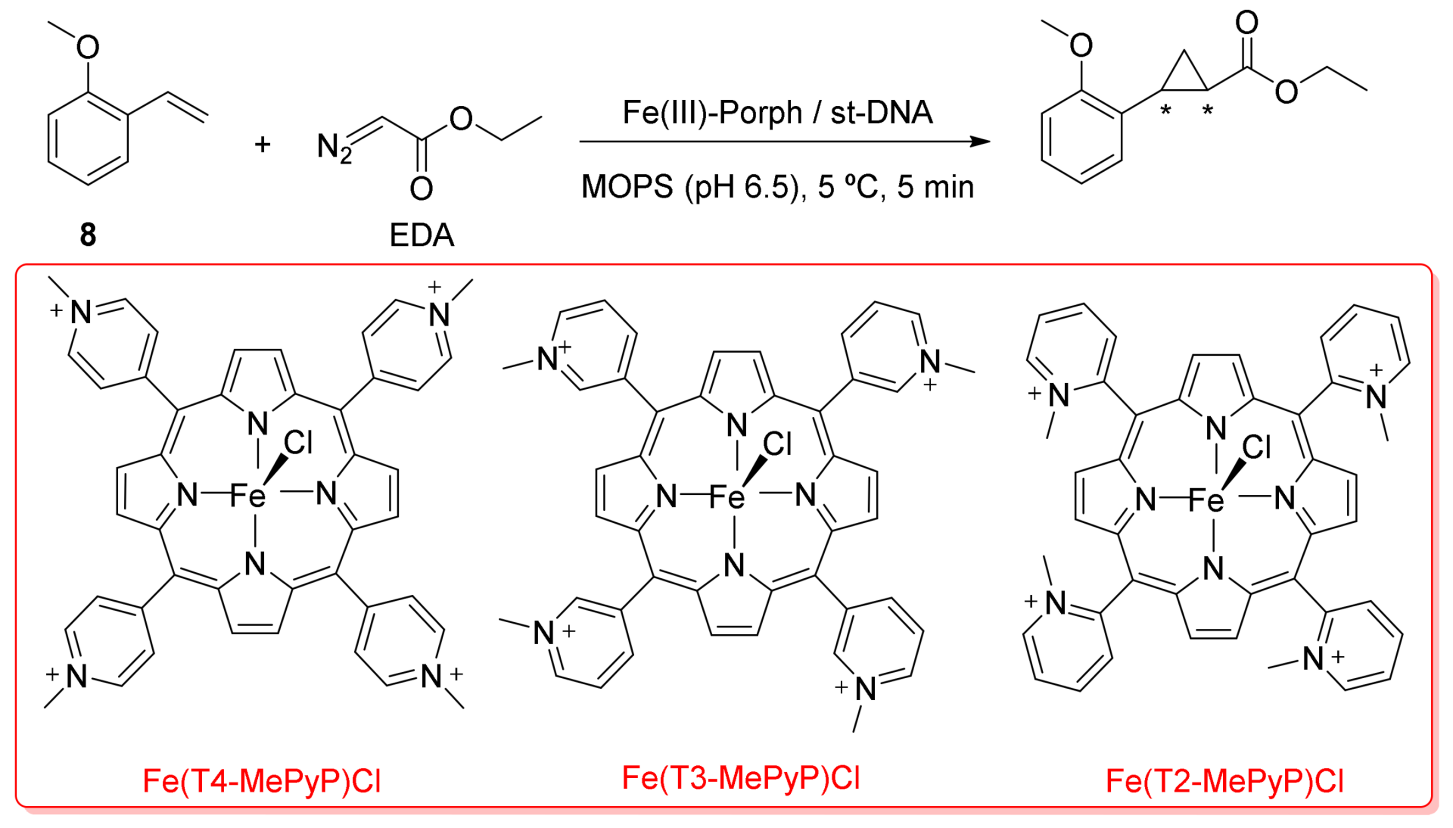
Cyclopropanation reaction of 2-methoxystyrene (**8**) with EDA catalysed by DNA/iron(III) *meso*-tetrakis(*N*-methylpyridinium-yl)porphyrin hybrid catalysts.

The cationic *meso*-tetrakis(*N*-alkylpyridyl)porphyrins are well-known DNA binders. In such compounds the position of the *N*-alkyl groups and the length of the alkyl chains have a key role in the interactions between such cationic porphyrins and DNA. The DNA-based catalysts were self-assembled by combining the iron(III) porphyrins with salmon testes DNA (st-DNA, 6 mM in base pairs) in 20 mM 3-(*N*-morpholino)-propanesulfonic acid (MOPS) buffer at pH 6.5, which was found to be the optimal pH value with regard to both activity and selectivity. The cyclopropanation of 2-methoxystyrene (**8**) with ethyl diazoacetate (EDA) as the carbene precursor was selected as the benchmark reaction at 5 °C. In the absence of DNA, the cyclopropanation reaction was sluggish and resulted in low product yields. In the presence of st-DNA, low activity was observed in the case of the *para* and *meta* porphyrin isomers, whereas with the *ortho* isomer immediate and vigorous evolution of N_2_ was observed, which ended after circa 5 min with total EDA consumption, although only 14% yield of the *trans* isomer was obtained, for 42% *ee* (Scheme 8). The scope of the cyclopropanation reaction was evaluated for 4-methoxystyrene, 4-chlorostyrene, and styrene with EDA and other diazo compounds. In all cases, a major amount of cyclopropanation product was obtained only in the presence of DNA [61].

**Scheme 9:**
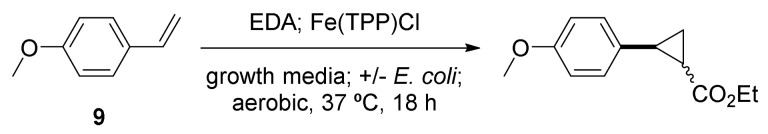
Cyclopropanation of 4-methoxystyrene (**9**) with EDA catalysed by Fe(TPP)Cl in aqueous media and in the presence or absence of *E. coli*.

Wallace and Balskus reported a biocompatible Fe(III) porphyrin catalyst system capable of efficient olefin cyclopropanation in the presence of a living microorganism at 37 °C under aerobic conditions. In fact, the Fe(TPP)Cl catalysed cyclopropanation of 4-methoxystyrene (**9**) with EDA occurred in aqueous media, both in the presence and in the absence of an engineered *E. coli*, affording analogous cyclopropane product yields and *trans/cis* ratios. Moreover, a similar Fe(III) phthalocyanine proved to be an even better catalyst for the cyclopropanation of 4-methoxystyrene (**9**) in the presence of the same *E. coli* affording the cyclopropane product in 95% yield. These preliminary studies proved the efficiency of olefin cyclopropanation under conditions compatible with the growth of *E. coli* [62]. Therefore, by interfacing the Fe(III) phthalocyanine with an *E. coli* engineered to produce styrene, the authors were able to synthesize non-natural phenyl cyclopropanes directly from D-glucose in a single-vessel fermentation (Scheme 9) [62].

An efficient and selective methodology for the cyclopropanation of various styrene derivatives with EDA, catalysed by tetraphenylporphyrinatotin(IV) trifluoromethanesulfonate [Sn^IV^(TPP)(OTf)_2_] and tetraphenylporphyrinatotin(IV) tetrafluoroborate [Sn^IV^(TPP)(BF_4_)_2_] was reported. Only pure *trans*-isomers were produced in excellent yields and short reaction times (17–40 min), for both catalysts, at ambient temperature, under nitrogen atmosphere, and using dichloromethane as solvent. Moreover, both catalysts were reused three times without loss of catalytic activity [63].

**Scheme 10:**
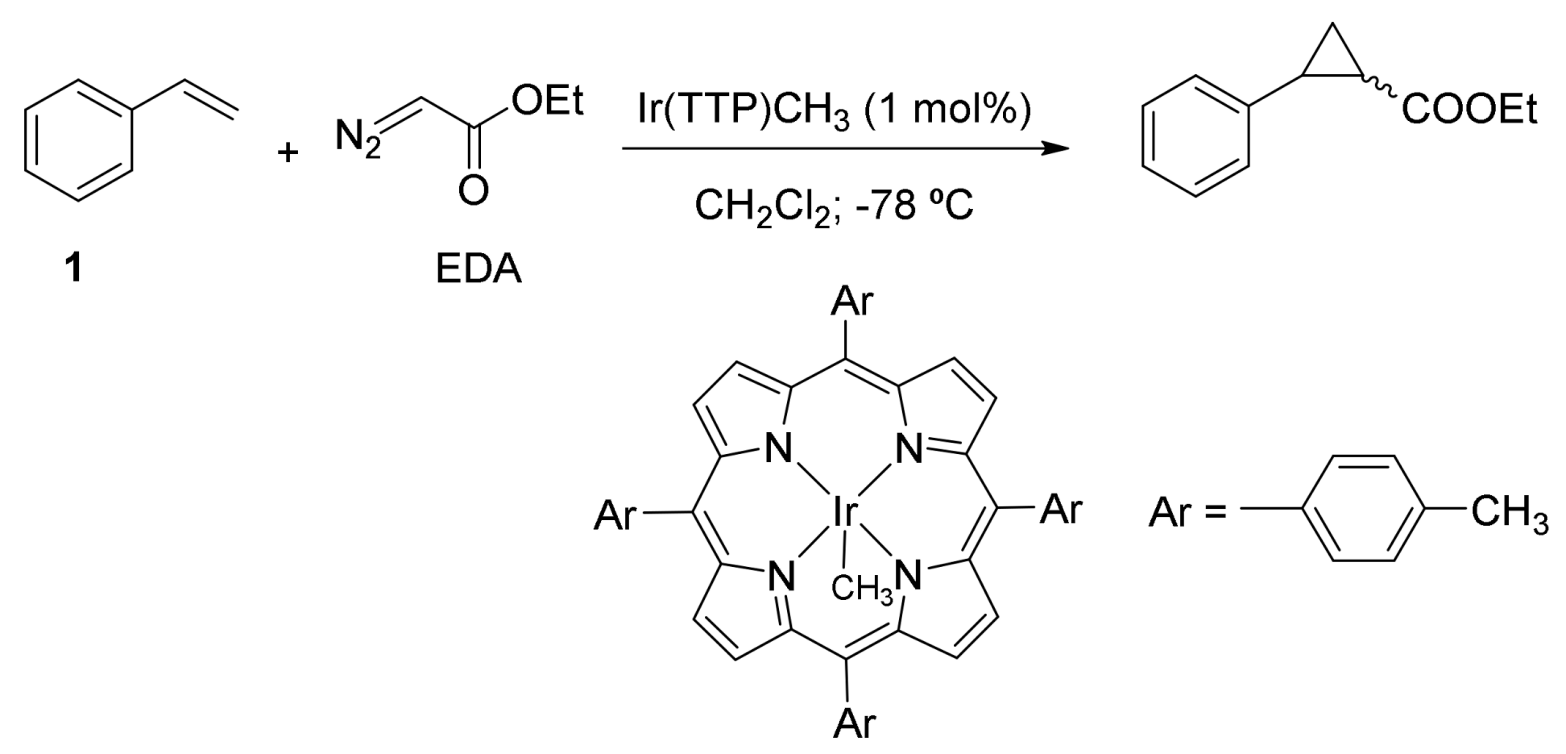
Cyclopropanation of styrene (**1**) with EDA catalysed by Ir(TTP)CH_3_.

Very recently, iridium(III) porphyrin complexes were described for the first time as active catalysts for carbene transfer reactions. Indeed, several iridium(III) tetratolylporphyrin (TTP) complexes showed to be extremely active and robust catalysts for the cyclopropanation of olefins using diazo compounds as carbene sources. In particular, Ir(TTP)CH_3_ (Scheme 10) catalysed the cyclopropanation of styrene (**1**) with ethyl diazoacetate (EDA) in CH_2_Cl_2_ at −78 °C achieving 85% yield in less than 5 min. At very low catalyst loadings, the styrene cyclopropanation reaction achieved 4.8 × 10^5^ TON in three successive EDA additions without significant catalyst deactivation. Yields of 93%, 92%, and 91% have been obtained for the first, second, and third additions. With other electron-rich and sterically unhindered substrates high yields and modest *trans* selectivities were obtained; however for electron-deficient and hindered olefins lower yields were obtained even in longer reaction times and higher temperatures [64].

**Scheme 11:**
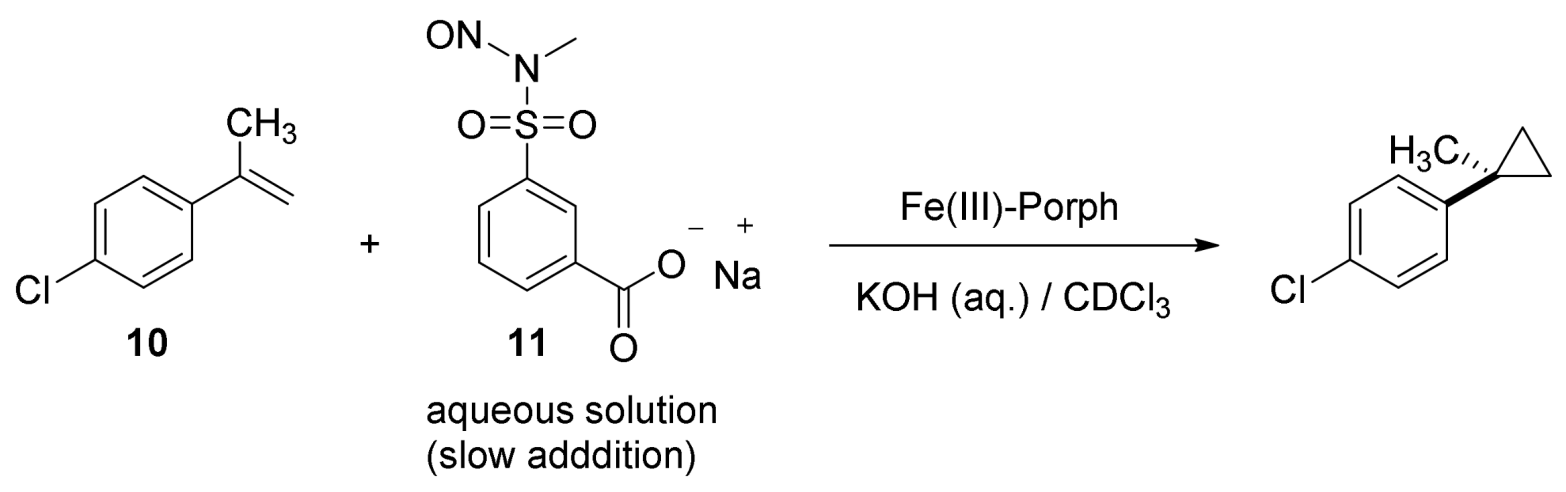
Cyclopropanation of styrene derivative [1-chloro-4-(prop-1-en-2-yl)benzene] (**10**) with sodium 3-(*N*-methyl-*N*-nitrososulfamoyl)benzoate (**11**) as the diazomethane precursor catalysed by Fe(III) porphyrin dendrimers.

**Figure 5:**
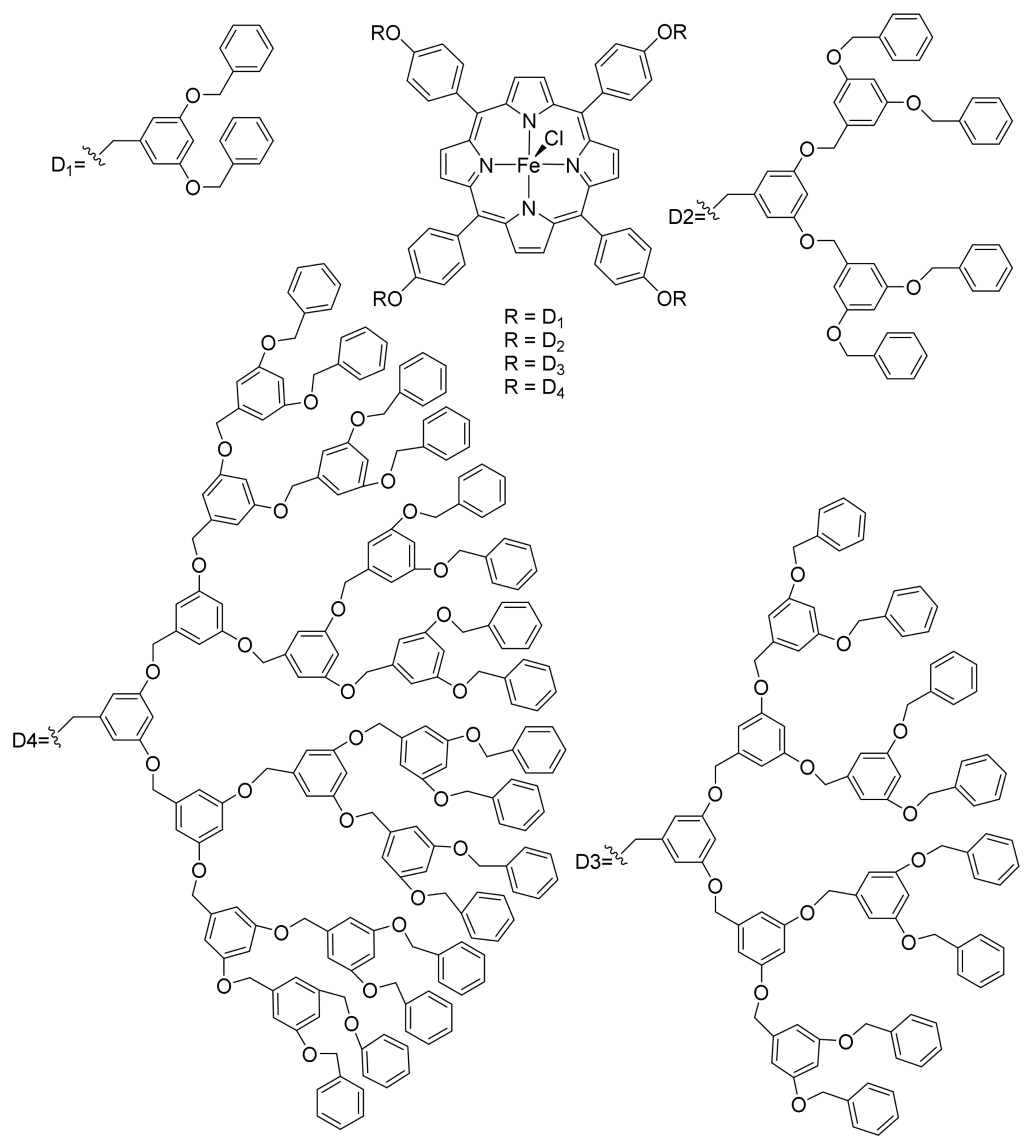
The dendrimeric metalloporphyrin catalysts derived from the 5,10,15,20-tetrakis(4-hydroxyphenyl)porphyrin under conventional Williamson etherification conditions.

Cossío and co-workers [65] have explored the catalytic properties of different generations of dendrimers based on Fe(III) porphyrin catalytic cores on the intermolecular cyclopropanation of substituted styrene [1-chloro-4-(prop-1-en-2-yl)benzene] (**10**) with diazomethane generated in situ, under basic conditions, from an aqueous solution of sodium 3-(*N*-methyl-*N*-nitroso-sulfamoyl)benzoate (**11**) slowly added by means of an automatic syringe. The reaction was performed in a biphasic system (water/CDCl_3_) (Scheme 11, Figure 5).

The reported symmetric dendrimers incorporate polyether dendritic arms around the 5,10,15,20-tetrakis(4-hydroxyphenyl)porphyrin moiety (Figure 5). The results showed that the cyclopropanation reaction progress was almost unchanged when the simpler metalloporphyrins (R = Me or Bn) were compared with Fe(TPP)Cl. The same profile was registered for the analogous dendrimers up to the second generation (R = D_1_, R = D_2_). However with third (R = D_3_) and fourth (R = D_4_) generations of catalyst dendrimers the reactions rate decreased significantly [65].

**Scheme 12:**
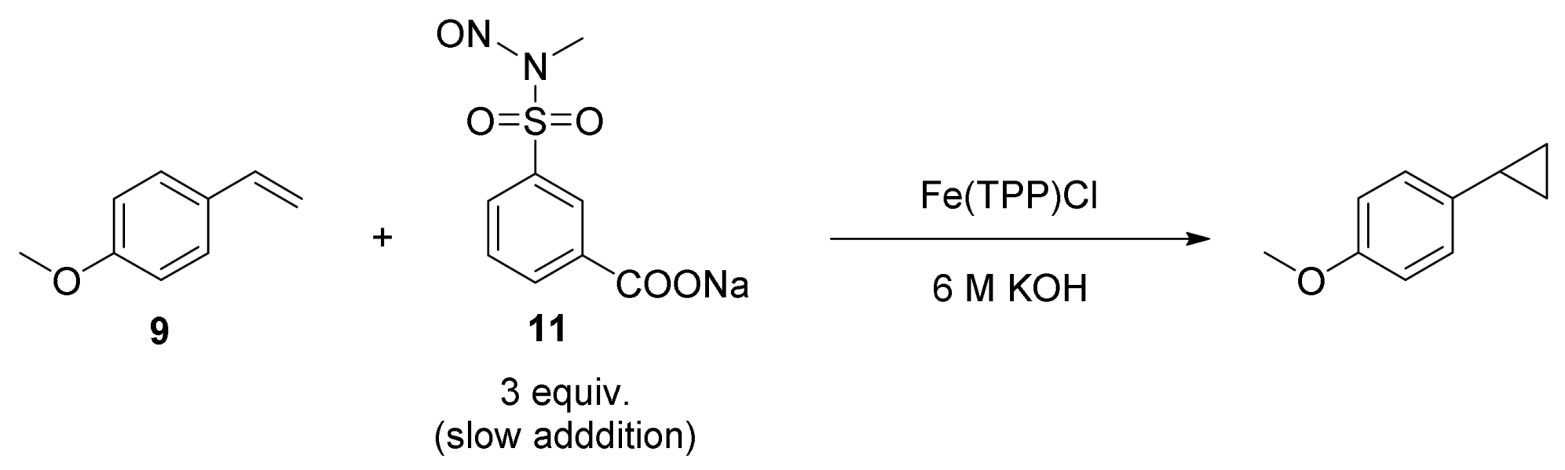
Cyclopropanation of *p*-methoxystyrene (**9**) catalysed by Fe(TPP)Cl with sodium 3-(*N*-methyl-*N*-nitrososulfamoyl)benzoate (**11**) as the diazomethane precursor, under two-phase conditions.

Morandi and Carreira [66] disclosed this safer use of diazomethane, obviating its isolation, purification, and handling, when conducting iron catalysed cyclopropanation reactions under strong alkaline conditions (aqueous 6 M KOH) in water. Moreover, the reactions were carried out in open air. The authors selected the cyclopropanation of *p*-methoxystyrene (**9**) to test the catalytic activity of several metal complexes under the demanding conditions required for the decomposition of the above-mentioned water-soluble sodium 3-(*N*-methyl-*N*-nitrososulfamoyl)benzoate (**11**), which was added slowly over 4 h to avoid any substantial build-up of diazomethane (Scheme 12). Fe(TPP)Cl (5 mol%) gave the highest conversion (100%), followed by Ru(TPP)CO and Co(TPP) with 93% and 33% conversions, respectively. Good yields were obtained for styrene derivatives containing electron-withdrawing and electron-donating substituents [66].

**Scheme 13:**
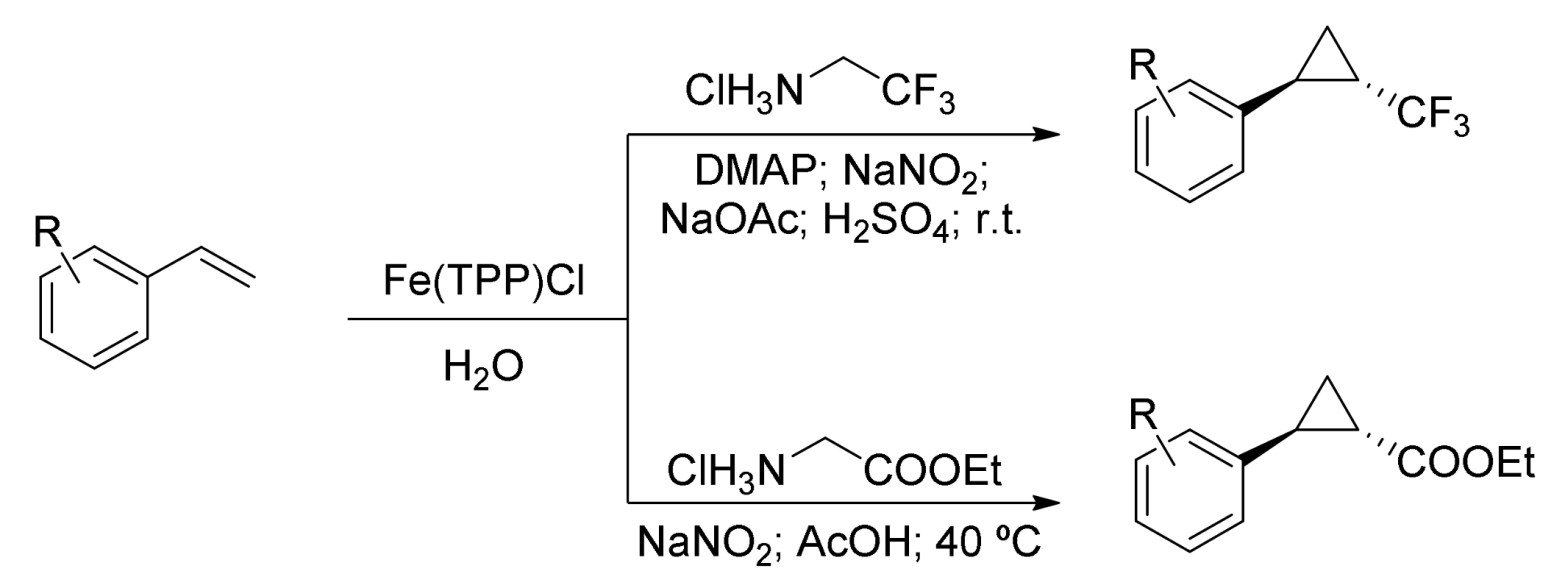
Iron(III) porphyrin catalysed cyclopropanation reaction of styrenes in aqueous media.

Furthermore, the Morandi and Carreira procedure can be used with trifluoromethyldiazomethane [67] or ethyl diazoacetate [68], generated in situ, for the transformation of olefins into vicinally substituted cyclopropanes. Water in the reaction media is needed for the preparation of the diazo compounds and the Fe(TPP)Cl catalyst acts under such conditions (Scheme 13). In fact there is the straightforward reaction of the carbene precursor that is generated by acid-catalysed diazotization, with the available substrate [69].

**Scheme 14:**
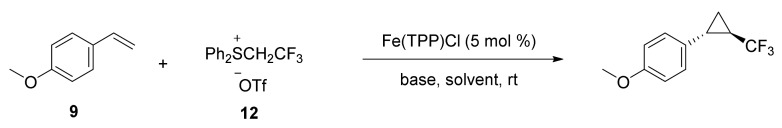
Fe(TPP)Cl catalysed cyclopropanation of 4-methoxystyrene (**9**) using (2,2,2-trifluoroethyl)diphenylsulfonium triflate (**12**) as the trifluoromethylcarbene precursor.

Trifluoromethylcarbene (:CHCF_3_) demonstrated to be a useful intermediate for the synthesis of CF_3_-containing compounds, being applied in several organic transformations, namely cyclopropanation, cyclopropenation, and aziridination. Generated from (2,2,2-trifluoroethyl)diphenylsulfonium triflate (Ph_2_S^+^CH_2_CF_3_^−^OTf) (**12**), the trifluoromethylcarbene was successfully applied in the cyclopropanation of several olefins catalysed by Fe(TPP)Cl, giving the corresponding trifluoromethylated cyclopropanes in good to high yields. Cu(II), Ni(II), and Co(II) complexes of TPP were inactive under similar reaction conditions. Depending on the solvent used, the base added, and the molar ratio tested (4-methoxystyrene:carbene precursor:base), the authors were able to obtain up to 88% yield after 4 h of reaction at ambient temperature (Scheme 14). Increasing the concentration of the starting materials and shortening the reaction time gave rise to comparable yields. Thus, the cyclopropanation of 4-methoxystyrene proceeded very fast affording the cyclopropane product in 85% yield within 30 min for a 1:1.1:1.2 molar ratio, using cesium fluoride as a base. Furthermore, the authors explored the scope of this trifluoromethylcarbene’s cyclopropanation catalysed by Fe(TPP)Cl under the optimized reaction conditions, where high *trans/cis* diastereoselectivity was observed in almost all the reactions (>98/2) [70].

**Scheme 15:**
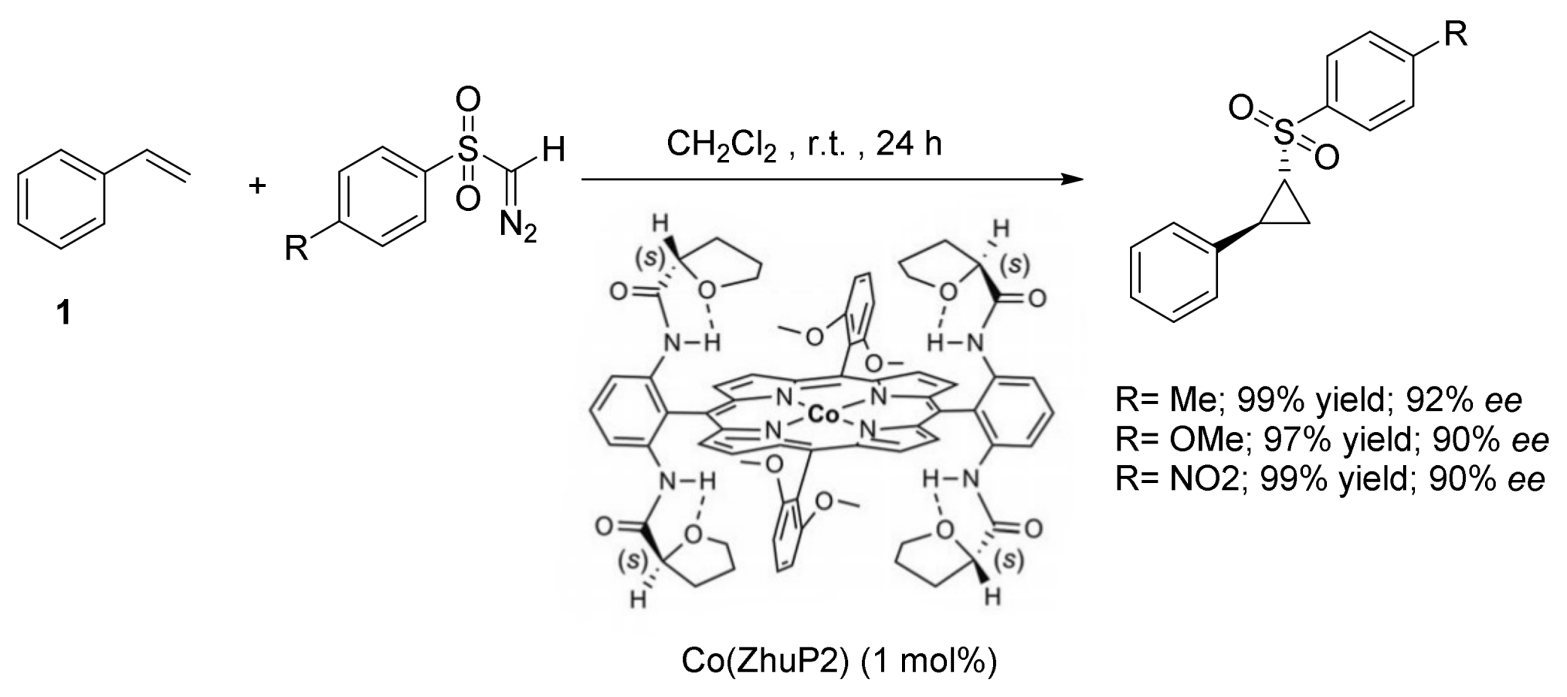
Asymmetric cyclopropanation of styrene (**1**) with diazosulfones catalysed by Co(ZhuP2) [71].

Zhang and co-workers [71] performed a study for the intermolecular cyclopropanation of styrene with diazosulfones catalysed by Co(II) chiral porphyrins. The so-called Co(ZhuP2) porphyrin has proved to be a general catalyst to enable the formation of enantiomerically enriched cyclopropyl sulfones from the reaction of a wide range of diazosulfones with alkenes (Scheme 15).

**Scheme 16:**
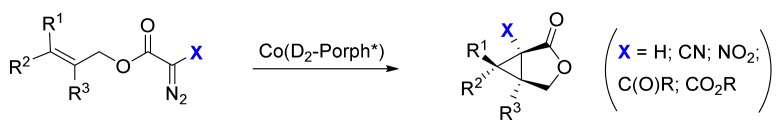
Asymmetric intramolecular cyclopropanation of allylic α-diazoacetates catalysed by Co(II) complexes of D_2_-symmetric chiral amidoporphyrins.

Zhang and co-workers had also referred the catalytically efficiency of Co(II) complexes of several D_2_-symmetric chiral amidoporphyrins in the asymmetric intramolecular cyclopropanation of allylic α-diazoacetates (X = H; CN; NO_2_; C(O)R; CO_2_R in Scheme 16) [72,73]. Since the resultant products possess three neighbouring chiral centres with numerous functionalities, this family of enantioselective transformations demonstrated to be highly important for synthetic applications, since product yields range from 51% to 99%, whereas the *trans/cis* ratio is almost exclusively 99:1, with *ee* values frequently higher than 90%. So, this metalloradical catalytic system seems to be suitable for cyclopropanation of allylic α-diazoacetates with several functional groups, generating bicyclic products with excellent diastereocontrol and good enantiocontrol [72,73].

**Scheme 17:**
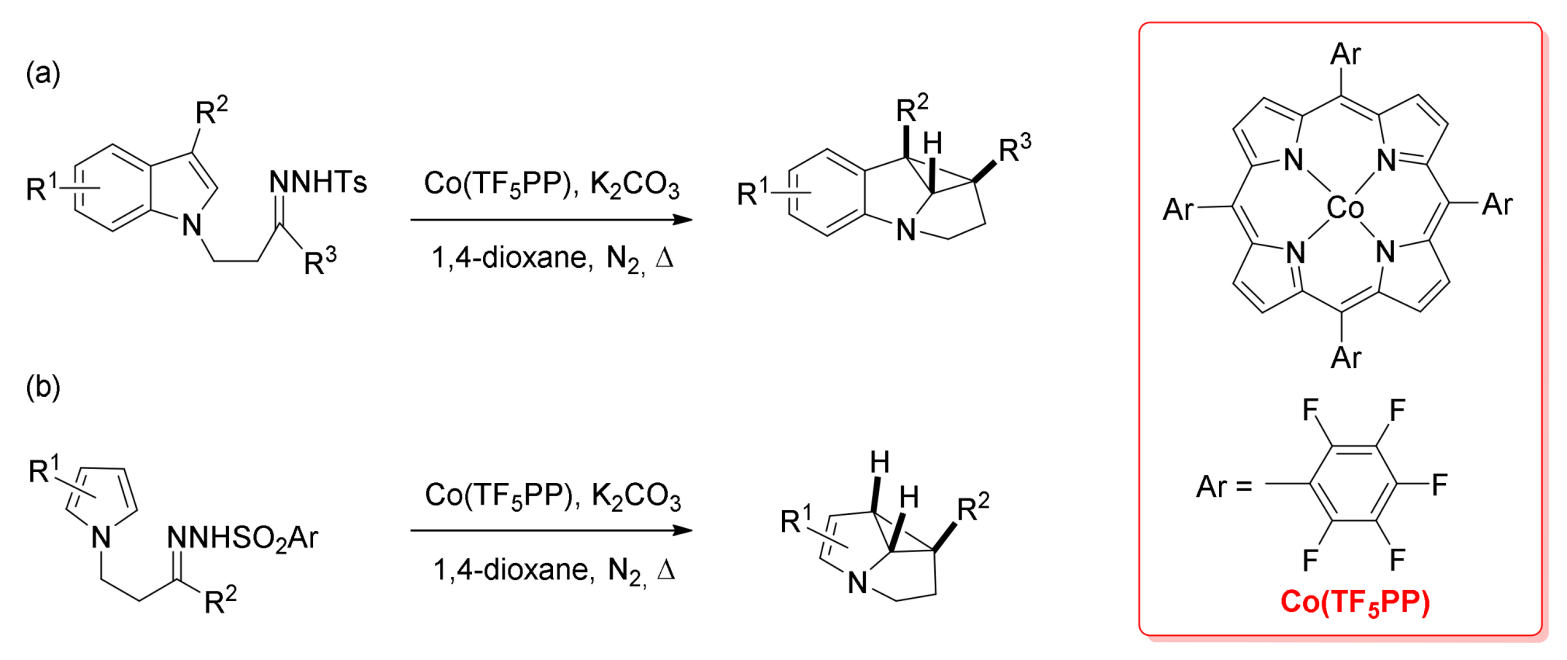
Co(II) porphyrin catalysed intramolecular cyclopropanation of (**a**) indolyl *N*-tosylhydrazones to form cyclopropane-fused indolines and (**b**) pyrrolyl *N*-arenesulfonylhydrazones to form cyclopropane-fused pyrrolines.

A highly chemoselective intramolecular cyclopropanation of *N*-alkyl indoles or pyrroles with alkyl diazomethanes, generated in situ from hydrazones, catalysed by Co(II) porphyrins, has been developed by Che and co-workers [74]. With the best catalyst, Co(TF_5_PP), a series of *N*-tosylhydrazones derived from several indoles gave rise to intramolecular cyclopropanation to afford the corresponding tetracyclic cyclopropane-fused indolines (Scheme 17a), whereas the intramolecular cyclopropanation of pyrroles led to the equivalent cyclopropane-fused pyrrolines (Scheme 17b) [74].

**Scheme 18:**
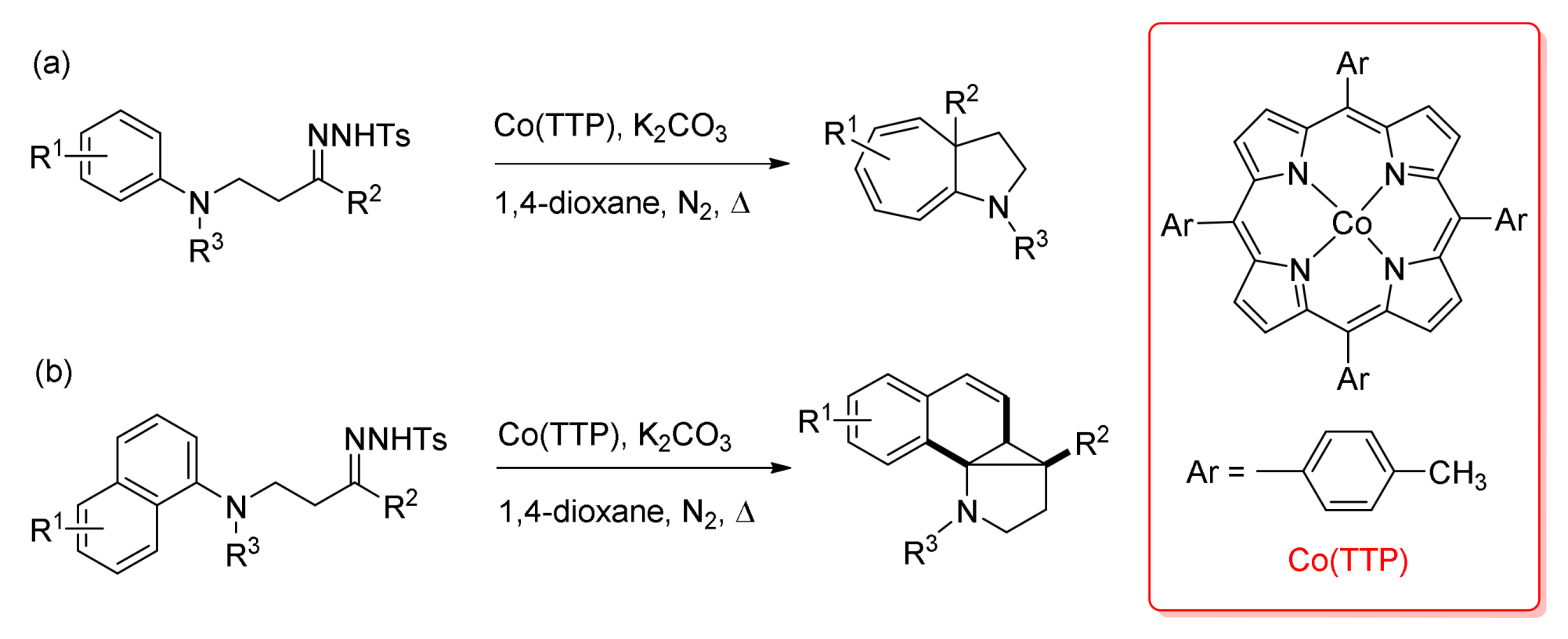
Co(TTP) catalysed intramolecular Buchner reaction of (**a**) aniline derived *N*-tosylhydrazones and (**b**) arene cyclopropanation of naphthylamine derived *N*-tosylhydrazones.

More recently, the same research group reported a Co(II) porphyrin catalysed intramolecular Buchner reaction and arene cyclopropanation of alkyl diazomethanes, generated in situ from *N*-tosylhydrazones, affording bicyclic cycloheptatriene-fused pyrrolidines and tetracyclic cyclopropane-fused pyrrolidines in good to high yields and with high chemo- and regioselectivities. Various *N*-tosylhydrazones derived from different anilines suffered intramolecular Buchner reactions giving cycloheptatriene-fused pyrrolidines in good to high yields (up to 95%). Moreover, the Co(II) catalysed Buchner reaction showed to be tolerant to different R^1^, R^2^ and R^3^ functional groups (Scheme 18a) [75].

Notably, when naphthylamine derived *N*-tosylhydrazones were used, in which amino substitution is at position C_1_ or C_2_ of naphthalene, different types of products were formed. Such as aniline derived *N*-tosylhydrazones, the C_2_ amino-substituted naphthylamine originated cyclopropanation with the less sterically hindered double bond of naphthalene (between C_2_-C_3_) and subsequent electrocyclic ring opening to give the Buchner cycloheptatriene product in high yield. On the other hand, the reaction of the C_1_ amino-substituted naphthylamines stopped at the cyclopropanation stage to give a tetracyclic compound in 47–99% yield, and no Buchner product was formed (Scheme 18b) [75].

**Scheme 19:**
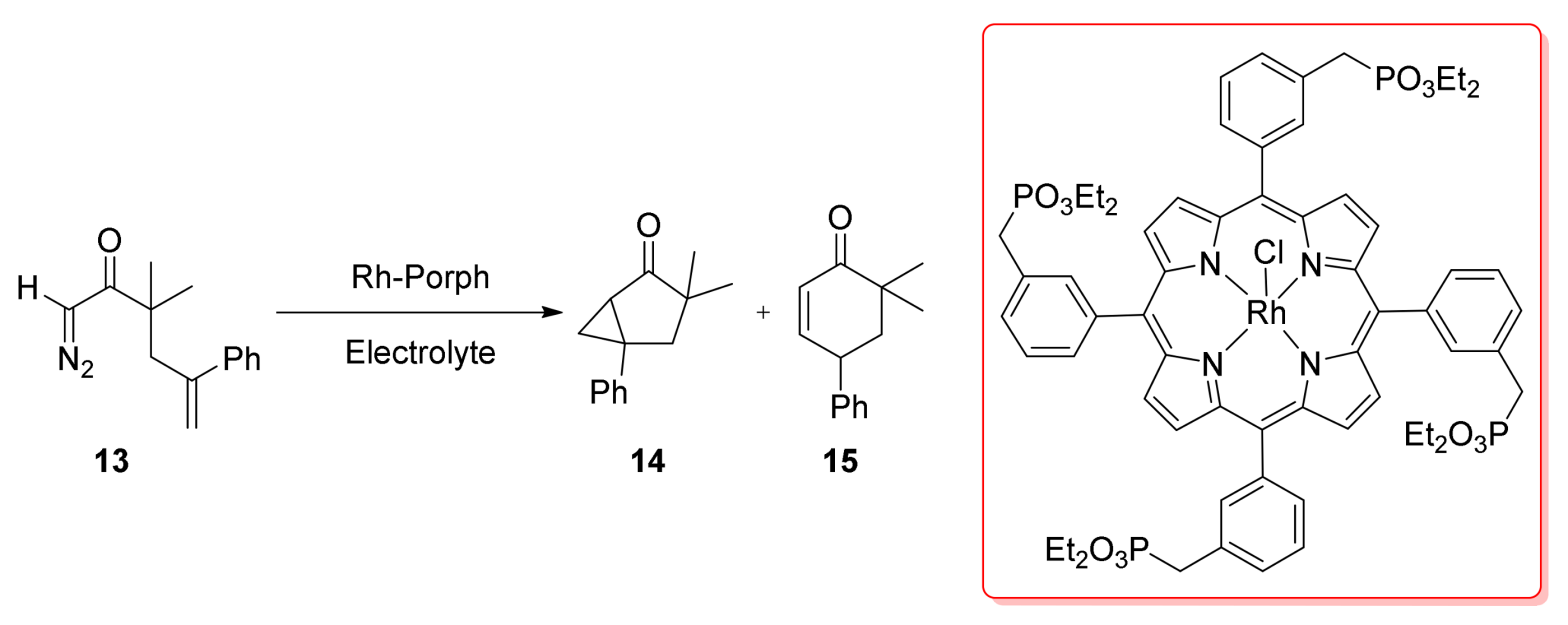
The intramolecular reaction catalysed by the Rh tetraarylporphyrin (with four phosphonate esters) that was covalently linked to the dielectric-electrolyte interface in the parallel plate cell.

An intramolecular reaction catalysed by Rh porphyrins was studied by Kanan and co-workers in the presence of interfacial electric fields. The reaction chosen for the electric field effect studies was a Rh-catalysed intramolecular carbene reaction using a diazoketone as the carbene precursor (Scheme 19). Using 1-diazo-3,3-dimethyl-5-phenylhex-5-en-2-one (**13**) and the Rh porphyrin the cyclopropanated product (**14**) and the inserted product (**15**) were obtained, presumably via a carbenoid intermediate [76]. To explore this reaction in the presence of an interfacial electric field, Si electrodes were covered with thin films of insulating (TiO_2_ or Al_2_O_3_) dielectric layers and were considered as opposite walls in the reaction container. The Rh porphyrin catalyst was placed at the dielectric-electrolyte interface. The product ratio was studied mainly as a function of the applied voltage. With no voltage applied, 10:1 was the ratio of (**14**):(**15**). Additionally, a voltage applied through a TiO_2_ surface gave rise to a Rh porphyrin-TiO_2_ interaction, which led to a (**14**):(**15**) ratio increase towards a maximum for which cyclohexenone (**15**) was nearly completely inhibited (>100:1), while the use of a dielectric with a Al_2_O_3_ surface resulted in a decrease in the (**14**):(**15**) ratio. So, these results demonstrated that the selectivity can be changed by subjecting the catalyst to an interfacial electric field [76].

The presence of physical electrodes might not be necessary in a reaction medium if a polarized interface can be introduced in it. This has been considered by Kanan and coworkers who used ball milled ferroelectric nanoparticles such as BaTiO_3_ in their studies on the Rh-catalysed intramolecular carbene reaction. Authors reported that if ferroelectric BaTiO_3_ nanoparticles are added to the reaction medium it can be observed a change in the product ratio of similar magnitude to the one observed in a reaction at an electrode-electrolyte interface polarized by an applied voltage [77].

**Figure 6:**
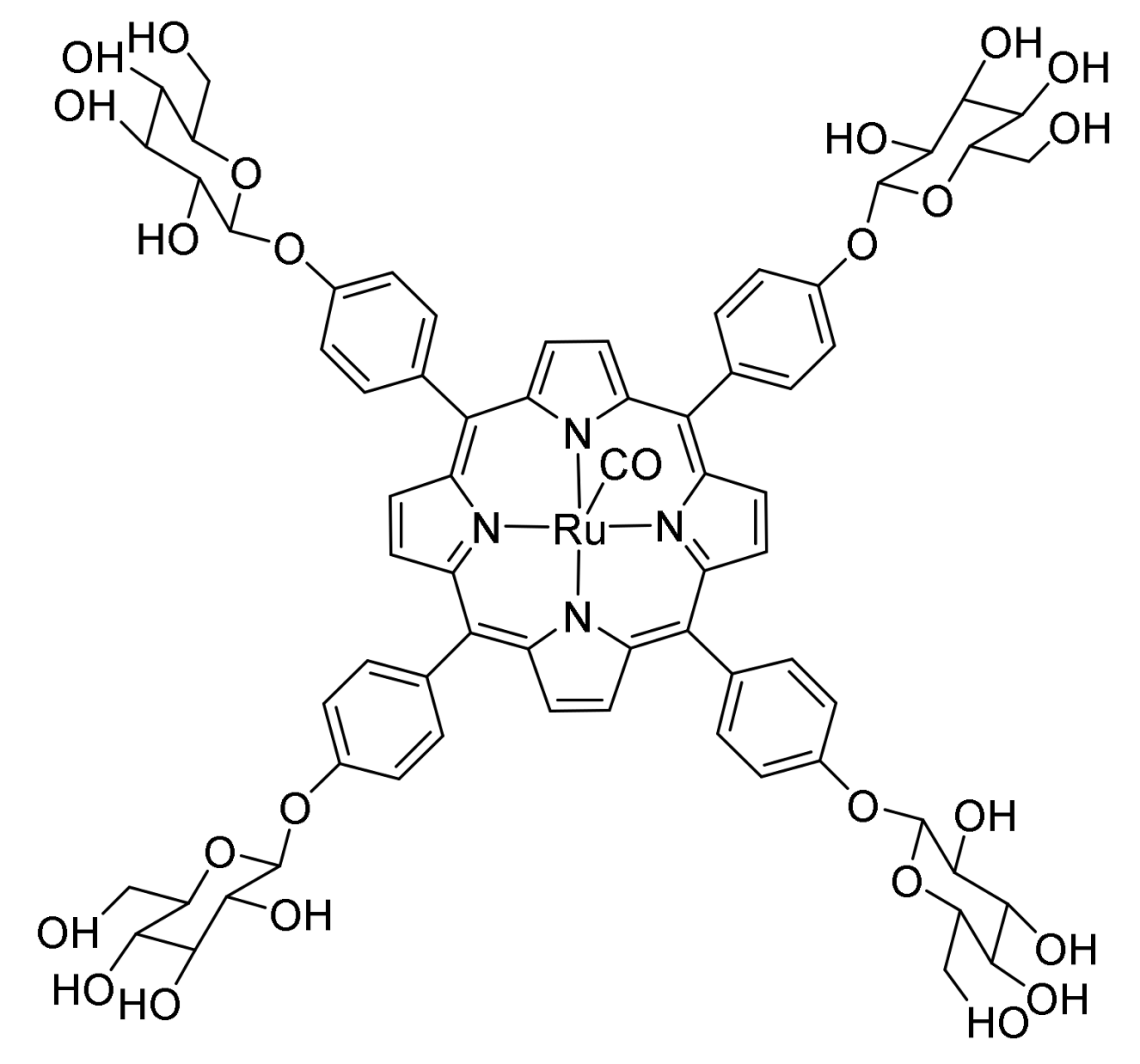
The water-soluble Ru(II) glycosylated porphyrin complex used as catalyst for carbenoid transfer reactions in aqueous media [Ru^II^(4-Glc-TPP)(CO)] (4-Glc-TPP = *meso*-tetrakis(4-(β-d-glucosyl)phenyl)porphyrinato dianion).

**Scheme 20:**
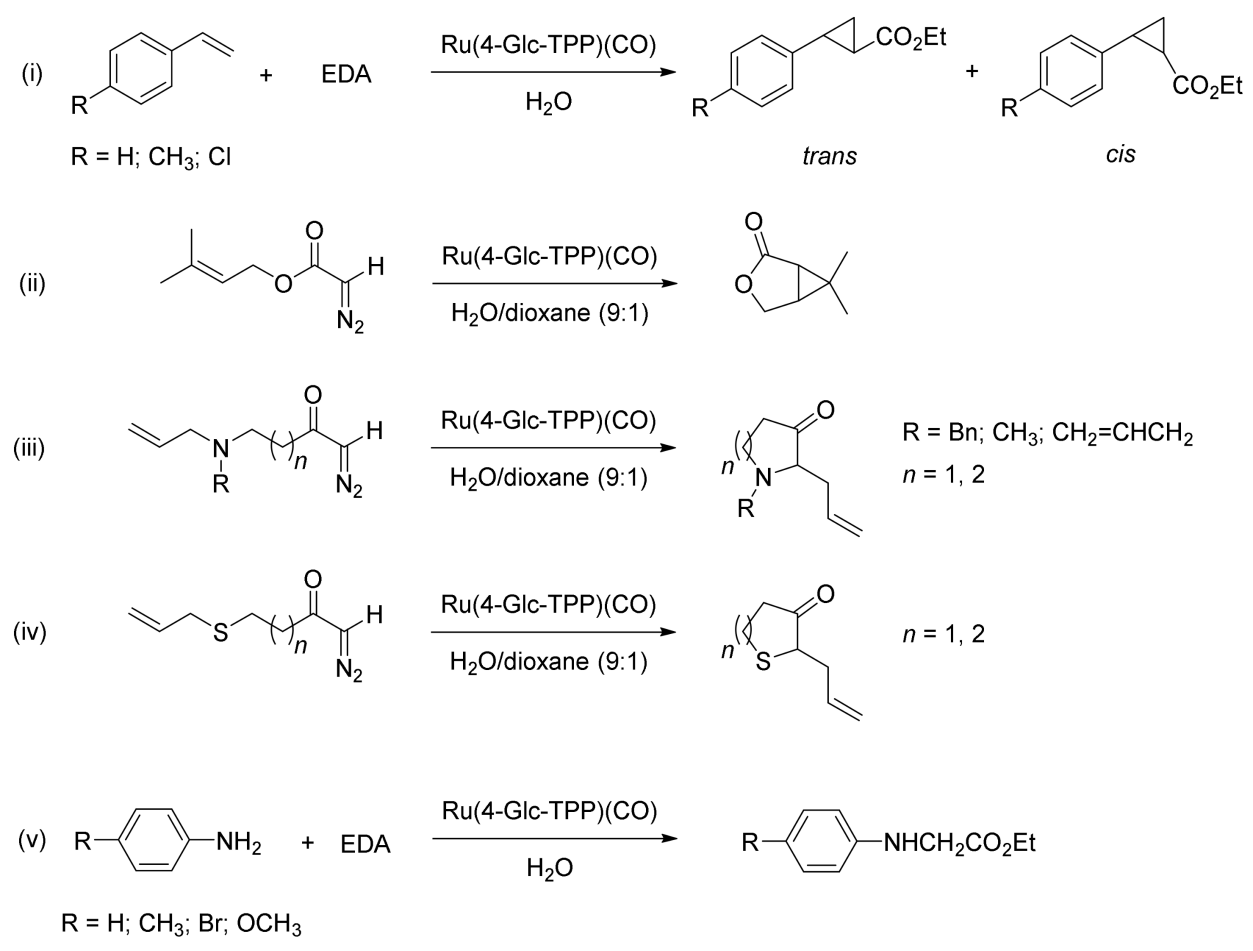
Reactions catalysed by the water-soluble Ru(II) glycosylated porphyrin complex.

Che and co-workers reported a water-soluble ruthenium(II) glycosylated porphyrin complex (Figure 6) that showed to be active as catalyst, in aqueous media, for (i) intermolecular cyclopropanation of 4-substituted styrenes (R = H, CH_3_, Cl) with EDA (70–76% yield; *trans/cis* 4–5:1); or (ii) intramolecular cyclopropanation of allylic diazoacetate (68% yield); or for (iii) diazoketones intramolecular ammonium ylide formation/[2,3]-sigmatroptic rearrangement (83–89% yield); or (iv) diazoketones intramolecular sulfonium ylide formation/[2,3]-sigmatroptic rearrangement (90–91% yield); and (v) intermolecular carbenoid insertion into N-H bonds of primary arylamines with EDA (76–83% yield). Conversion values which were obtained were 85–92% for styrene derivatives’ cyclopropanation (Scheme 20) [78].

## 3. Cyclopropenation Reactions

**Scheme 21:**
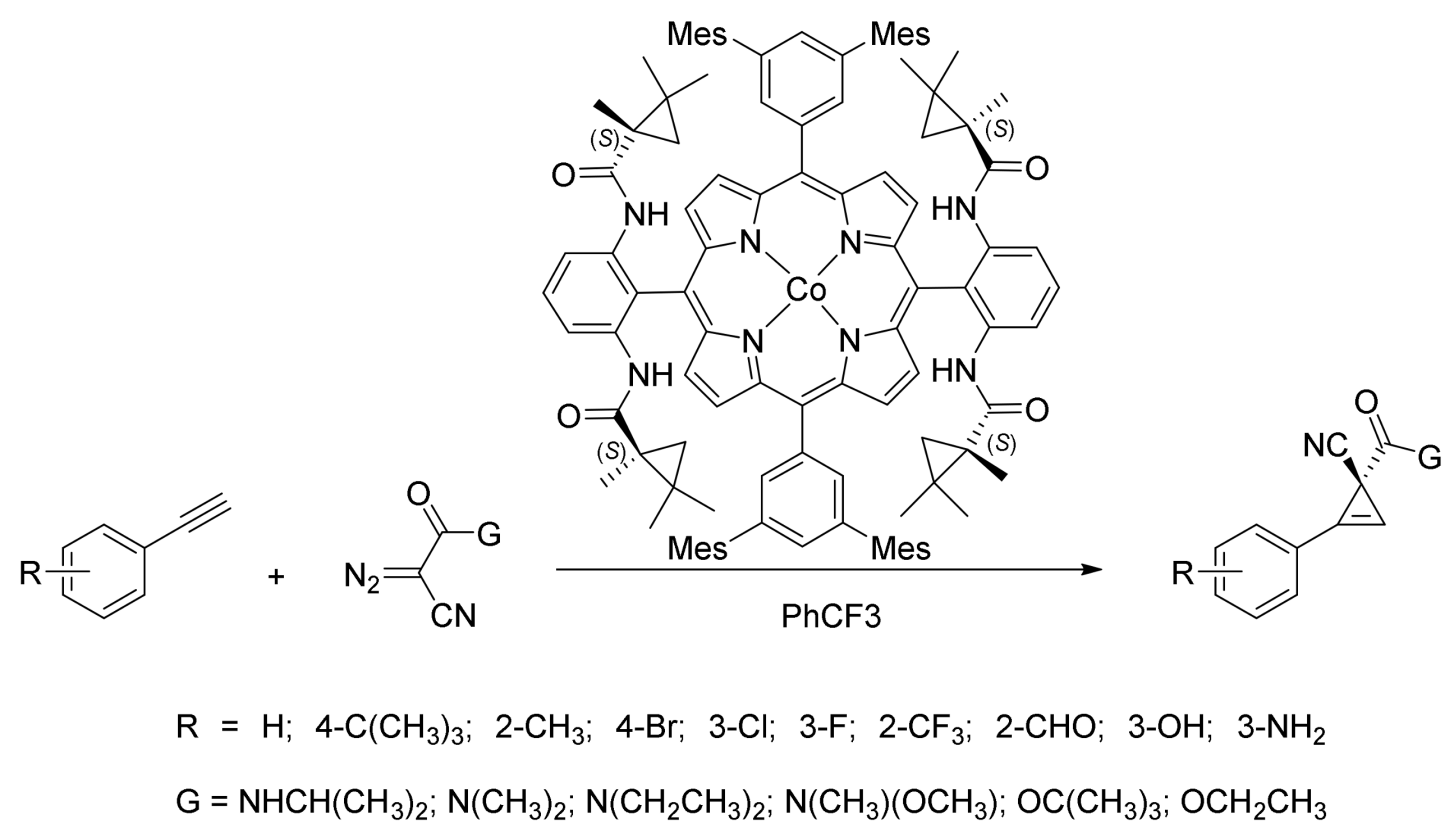
Asymmetric cyclopropenation catalysed by a Co(II) chiral porphyrin.

Cyclopropenes are a distinctive class of cyclic compounds with unsaturated, highly strained three-membered ring structures. The combination of this high strain with the unsaturation renders cyclopropenes as useful synthons for a range of subsequent organic transformations [79]. Some years ago, Zhang and co-workers reported the enantioselective cyclopropenation of alkynes with α-cyanodiazoacetates and α-cyanodiazoacetamides catalysed by a Co(II) complex of a D_2_-symmetric chiral porphyrin (Scheme 21). The reaction afforded strained cyclic products in high yield (up to 97% for R = 4-Br) and high enantioselectivities (80–99% *ee*), trifluorotoluene being the solvent of choice. This methodology proved to be valuable even for the synthesis of highly functionalized cyclopropenes having quaternary stereogenic centers [28,80]. We believe this is the only published work dealing with the cyclopropenation of alkynes catalysed by metalloporphyrins.

## 4. Carbene Insertion into C(sp^3^)-H

Direct functionalization of inactivated C-H bonds, particularly C(sp^3^)-H bonds, have attracted substantial attention in recent years. Despite the great efforts devoted to the field, the intermolecular C(sp^3^)-H bond activation of alkanes still remains a research target motivated by the inertness nature of simple aliphatic C(sp^3^)-H bonds. In this context, the catalytic carbene C(sp^3^)-H insertion stands as an important methodology for such purpose [81]. After the pioneering work by Callot and Metz, who reported that Rh(III) porphyrins were able to catalyse the intermolecular carbenoid insertion into C-H bonds of linear alkanes with ethyl diazoacetate (EDA), thus providing the first examples of carbene C-H insertion catalysed by a metalloporphyrin [82], several publications appeared in the literature involving mainly osmium, iron, copper, silver, rhodium or ruthenium porphyrins as catalysts for both inter- and intramolecular carbene insertion into C(sp^3^)-H [33,34]. However, due to the fact that these and other older publications have already been reviewed elsewhere [79,81], only the most recent works will be reviewed here.

**Scheme 22:**
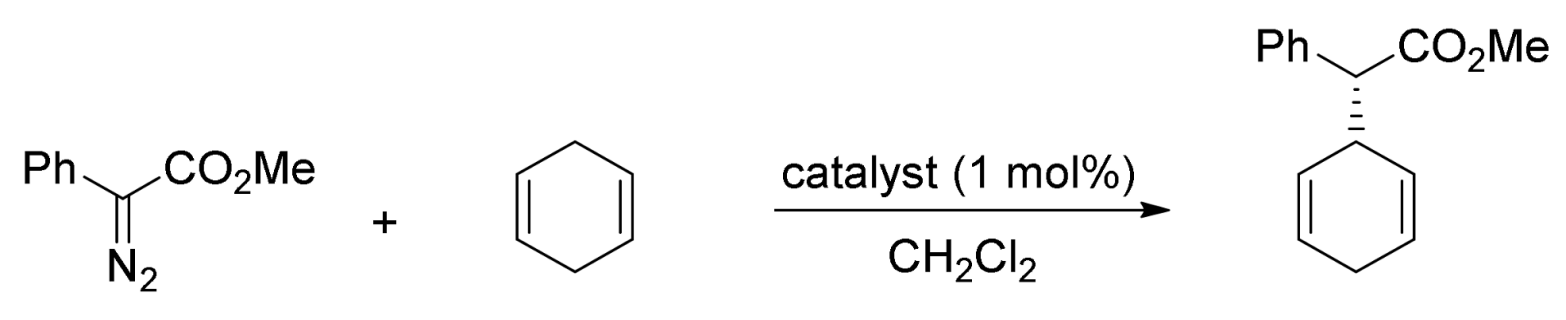
Intermolecular C-H insertion reactions of 1,4-cyclohexadiene with methyl phenyldiazoacetate catalysed by iridium(III) porphyrins.

**Figure 7:**
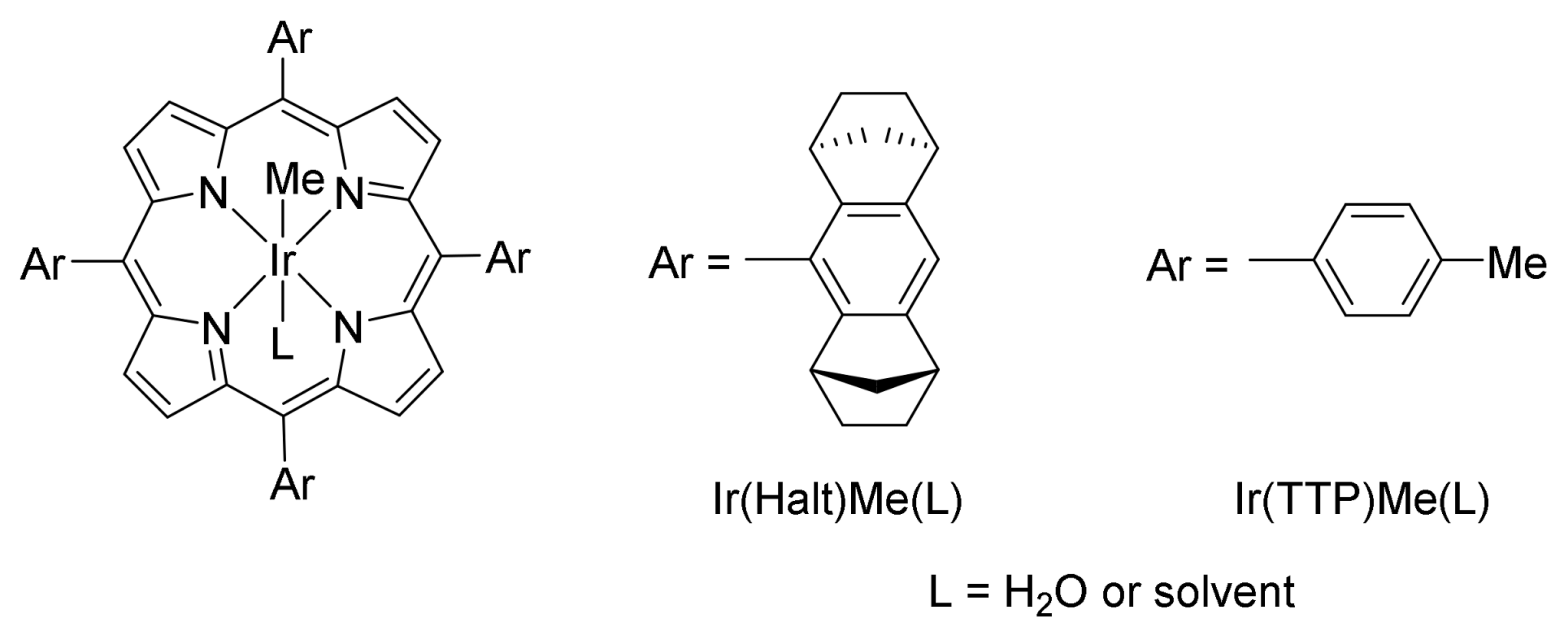
Chiral iridium(III) porphyrins used for catalysed carbene C-H and Si-H bond insertion reactions.

The transition metal catalysed decomposition of diazoesters for inter- and intramolecular carbene C-H insertion has been studied by several groups, namely by Che and co-workers [83,84]. With the complexes Ir(TTP)Me(L) and Ir(Halt)Me(L) (Figure 7), this research group examined their reactivity towards intermolecular carbene C-H insertion of 1,4-cyclohexadiene (**16**) with methyl phenyldiazoacetate (**17**) (Scheme 22). The reaction of 1,4-cyclohexadiene with methyl phenyldiazoacetate in the presence of 1 mol% Ir(TTP)(CO)Cl, at ambient temperature during 24 h, produced the C-H insertion product in 35% yield and 61% of conversion. A similar reaction with Ir(TTP)Me(L) was complete after 5 min and in 90% yield. This is obviously related with the higher activity of Ir(TTP)Me(L) when compared with Ir(TTP)(CO)Cl.

On the other hand, when the chiral catalyst Ir(Halt)Me(L) was used, the insertion product was obtained in 90% yield and 91% *ee*. Lowering the reaction temperature to −40 °C and extending the reaction time to 16 h led to a 95% *ee* for the same yield. When the reaction was performed at −78 °C a 93% *ee* was obtained after 24 h with similar yield. It is noteworthy that the carbene C-H insertion catalysed by the Ir(Halt)Me(L) was performed in a one-pot fashion and neither a dimer nor a cyclopropanation product were found in the reaction mixture [83].

**Scheme 23:**
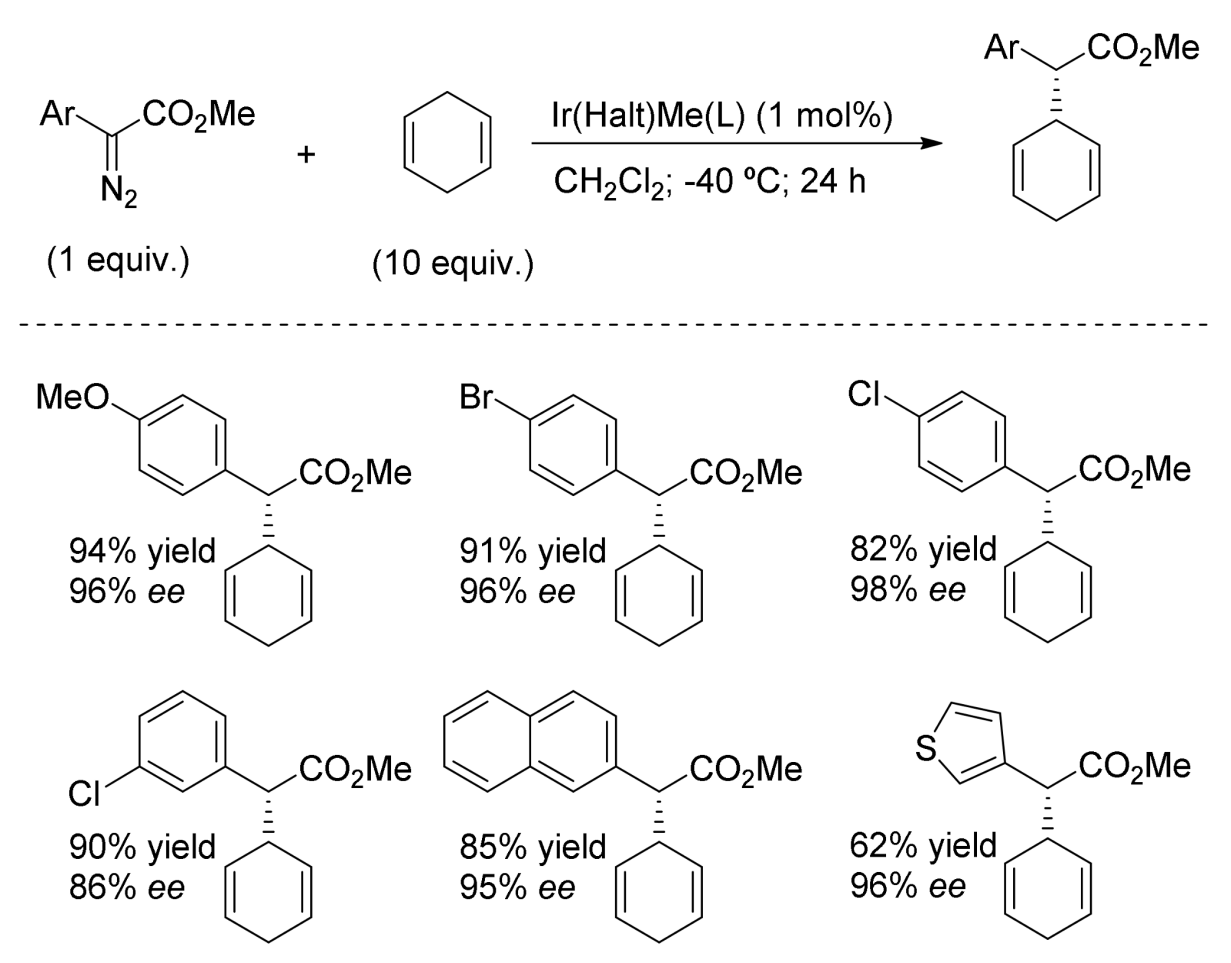
Intermolecular C-H insertion reactions of 1,4-cyclohexadiene with methyl aryldiazoacetate catalysed by the chiral porphyrin Ir(Halt)Me(L).

With the optimized conditions for the chiral porphyrin Ir(Halt)Me(L), Che and co-workers looked at the catalyst performance in catalysing C-H carbene insertion. The authors carried out the insertion of methyl α-aryl-α-diazoacetate into 1,4-cyclohexadiene; as a result they concluded that both electron-withdrawing and electron-donating groups at the *para* position of the diazoester aromatic ring did not suffer any transformation. A chlorine *meta*-substituted α-aryl-α-diazoacetate also gave the expected product in good yield and enantioselectivity; however with the corresponding *ortho*-chloro-substituted aryl substituted diazoacetate no reaction took place (Scheme 23) [83].

**Scheme 24:**
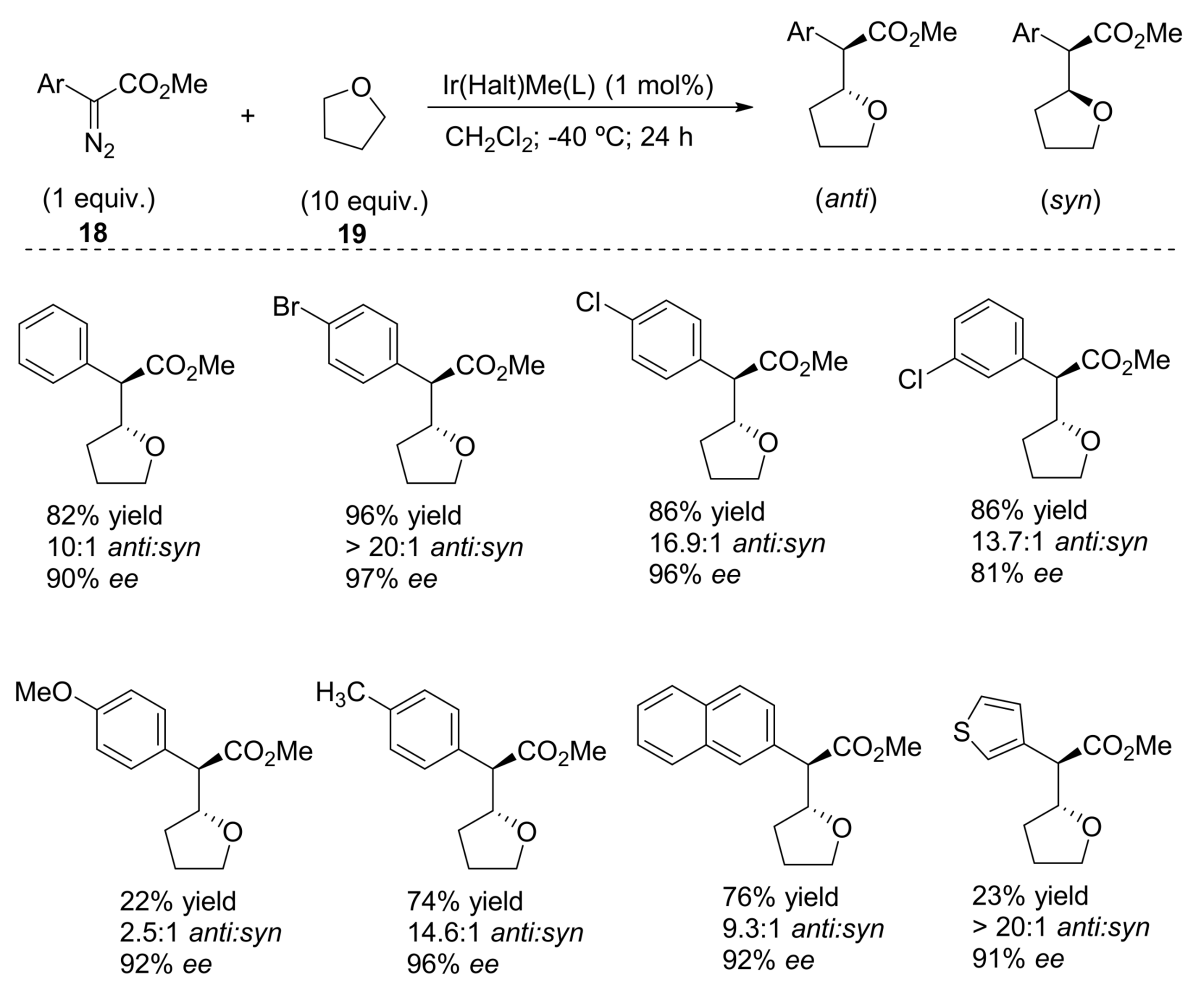
Intermolecular C-H insertion reactions of THF (**19**) with methyl aryldiazoacetate (**18**) catalysed by the chiral porphyrin Ir(Halt)Me(L).

The Ir(Halt)Me(L) catalysed C-H insertion of tetrahydrofuran (THF) by methyl aryldiazoacetate was also considered by the same authors in one-pot conditions. The reaction took place in dichloromethane, at −40 °C, and *anti* and *syn* C-H insertion products in 82% total yield with an *anti/syn* ratio of 10:1. The main *anti* isomer was obtained with an enantiomeric excess of 90% (Scheme 24). In general, all the tested aryl substituted diazo compounds produced C-H insertion products in good to high yields, high diastereoselectivities (up to *anti/syn* = 20:1) and enantioselectivities (up to 97% *ee*). The electron-donating methoxy substituent afforded low yield and diastereoselectivity, along with high enantioselectivity for the *anti*-isomer [83].

**Scheme 25:**
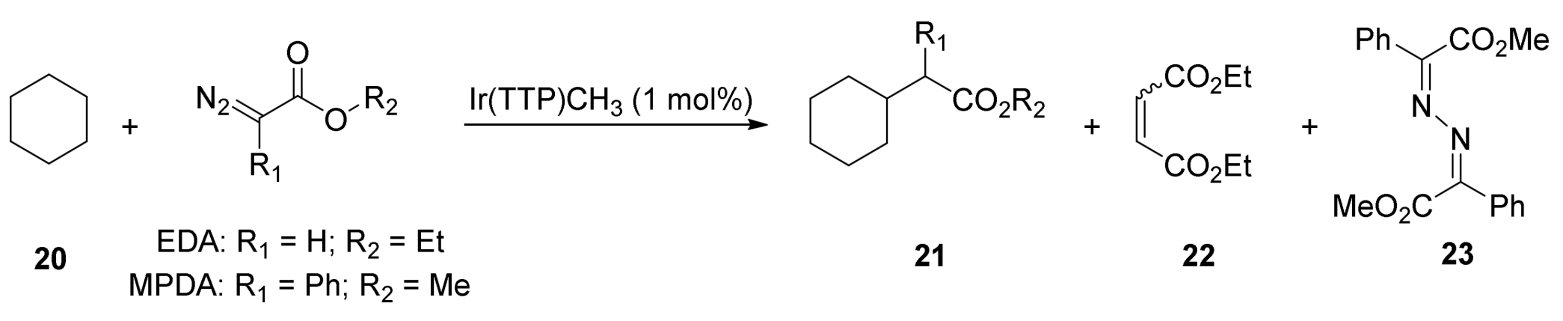
Intermolecular C-H insertion reactions of cyclohexane (**20**) with EDA and MPDA catalysed by Ir(TTP)CH_3_.

Woo and co-workers explored the C-H insertion reactions in neat cyclohexane (**20**) with 1.0 mol% Ir(TTP)CH_3_ (Scheme 25). Unluckly, the primary reactions using EDA did not produce the anticipated product (**21**). In its place, only the dimerization of the diazo reagent was observed, affording diethyl fumarate and diethyl maleate (**22**). By lowering the reaction temperature as an attempt to diminish that dimerization the reaction also failed. Presumably that can be attributed to the low solubility of Ir(TTP)CH_3_ in cyclohexane (**20**).

Considering that methyl phenyldiazoacetate (MPDA) is less reactive to dimerization, the authors changed their focus to the use of this reagent. The desired C-H insertion products were obtained, at room temperature, in low yields, being azine (**23**) the major product of the reaction formed by MPDA dimerization. Using higher temperatures and dropwise addition of the MPDA, and also increasing the catalyst loading to 5.0 mol% better yields of the insertion product (**21**) were obtained. However such a high loading is not adequate, taking into account the high price of Ir(TTP)CH_3_. Efforts to achieve the C-H insertion reaction with Ir(TTP)Cl(CO) as catalyst produced only trace quantities of (**21**). By placing Ir(TTP)CH_3_ to react with MPDA in pentane at reflux afforded (**23**) in almost quantitative yields. Also when using boiling octane, C-H insertion products were obtained in 37% yield. The insertion took place at the C_2_ and C_3_ secondary carbons (18%), and the primary insertion (7%) also took place. By outspreading this process to toluene and 1,4-cyclohexadiene, good yields were obtained for the equivalent benzylic and allylic C-H insertion products, respectively. It was also demonstrated that tetrahydrofuran is an appropriate substrate for C-H insertion [85].

**Scheme 26:**
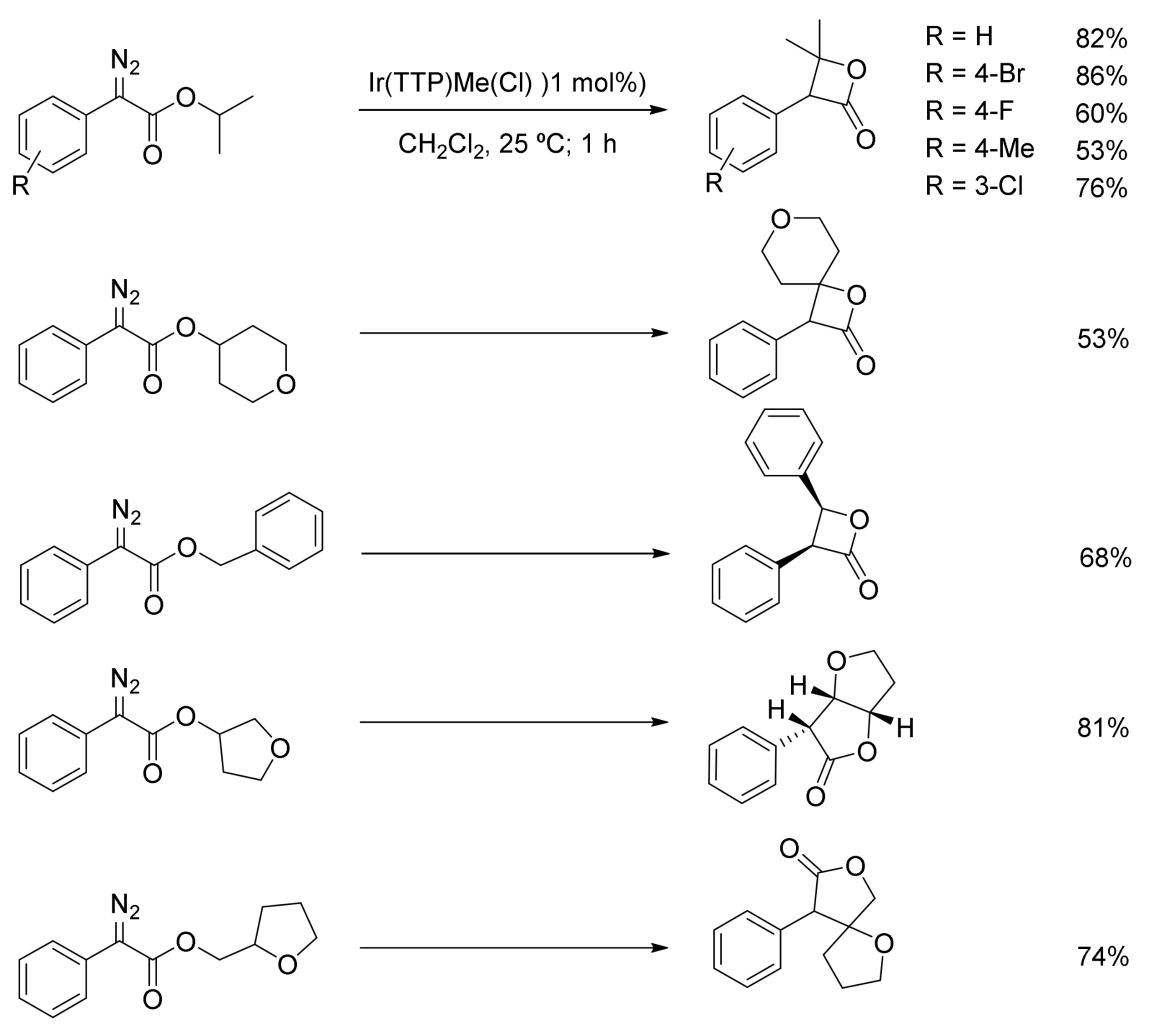
Intramolecular C-H insertion of α-diazoester catalysed by the achiral porphyrin Ir(TTP)Me(L).

Later, Che and co-workers demonstrated that Ir(TTP)Me(L) and Ir(Halt)Me(L) (Figure 7) are also capable of intramolecular carbene insertion into saturated C-H bonds of α-diazoesters, specifically leading to the generation of β-lactones in moderate to good yields. In particular, the achiral iridium(III) porphyrin complex Ir(TTP)Me(L) was found to be catalytically active towards the intramolecular carbene C-H insertion of α-diazoesters, giving exclusively the corresponding β-lactones (if carbene insertion to tertiary C-H bond occurs) or γ-lactones (if carbene insertion to secondary C-H bond occurs), in yields between 53% and 86% (Scheme 26) [84].

**Scheme 27:**
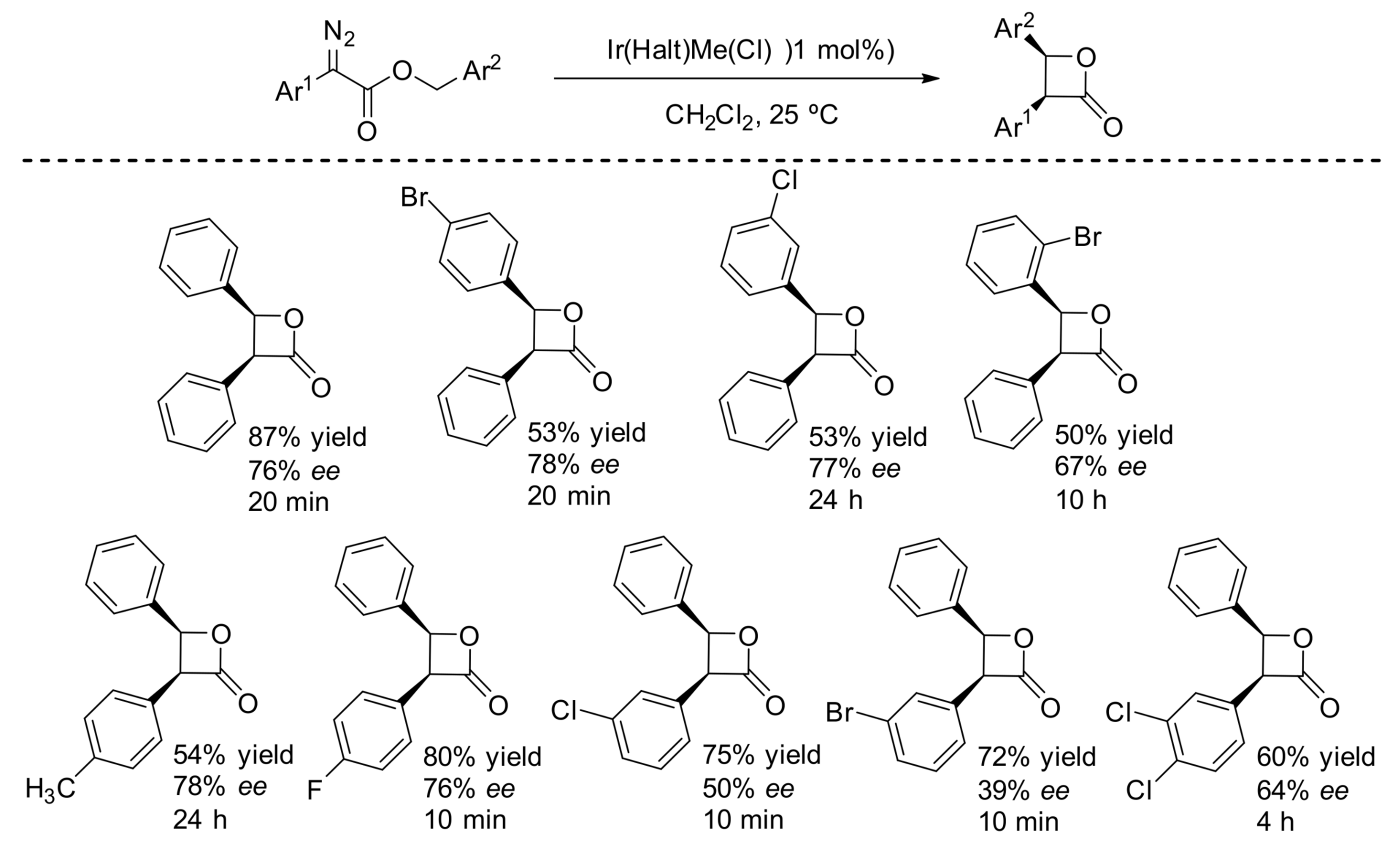
Intramolecular C-H insertion of α-diazoester catalysed by the chiral porphyrin Ir(Halt)Me(L). The isolated yields are presented.

In the same work, the authors also studied the asymmetric intramolecular carbene C-H insertion, catalysed by the chiral porphyrin Ir(Halt)Me(L). The intramolecular carbene C-H insertion of the α-diazoester (Ar_1_ = Ph and Ar_2_ = Ph) was selected as the model reaction for optimization of the reaction conditions. By treating the substrate with 1 mol% of the chiral catalyst, Ir(Halt)Me(L), in dichloromethane at 25 °C for 1 h, a 90% yield of the corresponding *cis*-β-lactone was found by ^1^H-NMR analysis of the crude reaction mixture (87% isolated yield with 76% *ee*). In order to explore the substrate scope of the reaction, several substrates with different Ar_1_ and Ar_2_ substitution patterns were tested and the corresponding *cis*-β-lactones were obtained in moderate to good isolated yields (up to 87%), excellent stereoselectivity (*cis*-products exclusively), and good enantioselectivities (up to 78% *ee*). (Scheme 27) [84].

**Figure 8:**
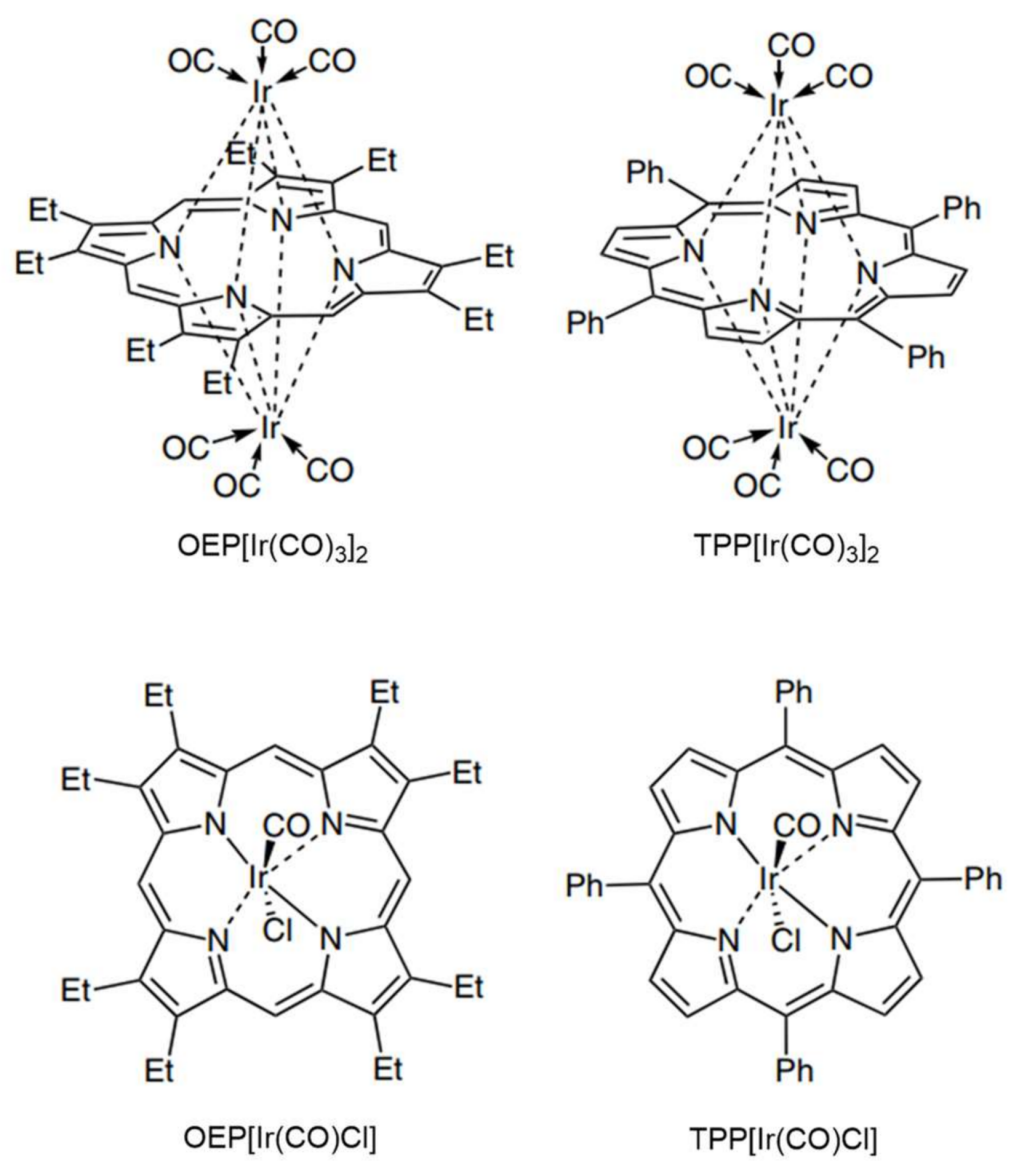
Octaethylporphyrin and tetraphenylporphyrin Ir(I) and Ir(III) complexes.

**Scheme 28:**
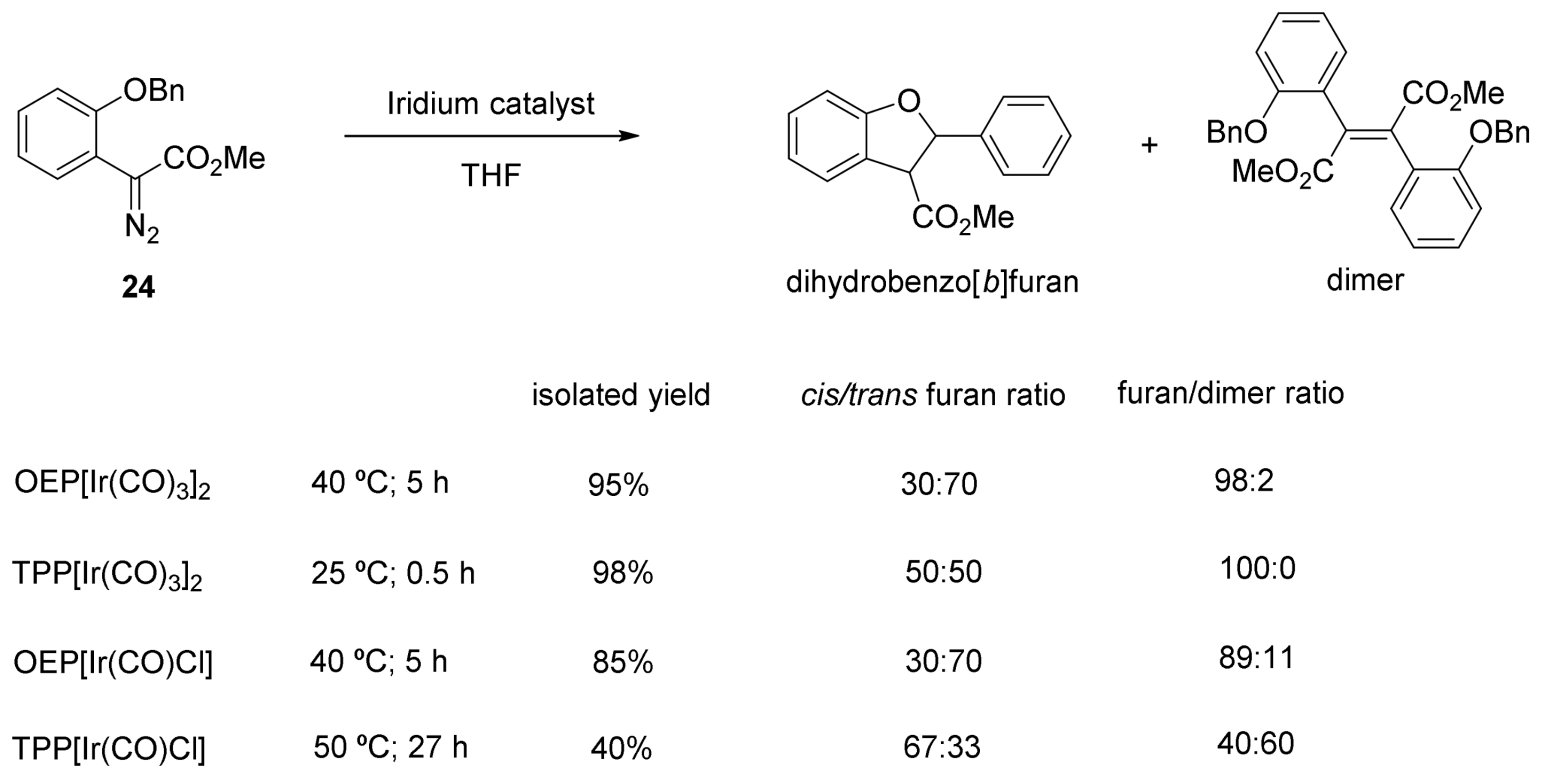
Intramolecular C-H insertion catalysed by octaethylporphyrin and tetraphenylporphyrin Ir(I) and Ir(III) complexes.

Octaethylporphyrin and tetraphenylporphyrin Ir(I) and Ir(III) complexes (Figure 8) showed to be useful catalysts for intramolecular C-H insertion processes of diazo compounds. The authors explored the reaction of 2-(2-benzyloxyphenyl)-2-diazoacetate (**24**) to obtain oxygenated five-membered rings through intramolecular C-H insertion reaction using iridium catalysts. While the Ir(I) complex TPP[Ir(CO)_3_]_2_ is the most efficient and selective catalyst, the others afford mixtures of cyclization and dimerization products (Scheme 28). The influence of the solvent was also studied using complex TPP[Ir(CO)_3_]_2_. While less polar solvents (toluene, THF, CH_2_Cl_2_) afforded only the cyclized product in excellent yields, more polar solvents (CHCl_3_, CH_3_CN) promoted also the formation of dimers. In DMSO, the dimerization product was formed exclusively [86].

**Scheme 29:**
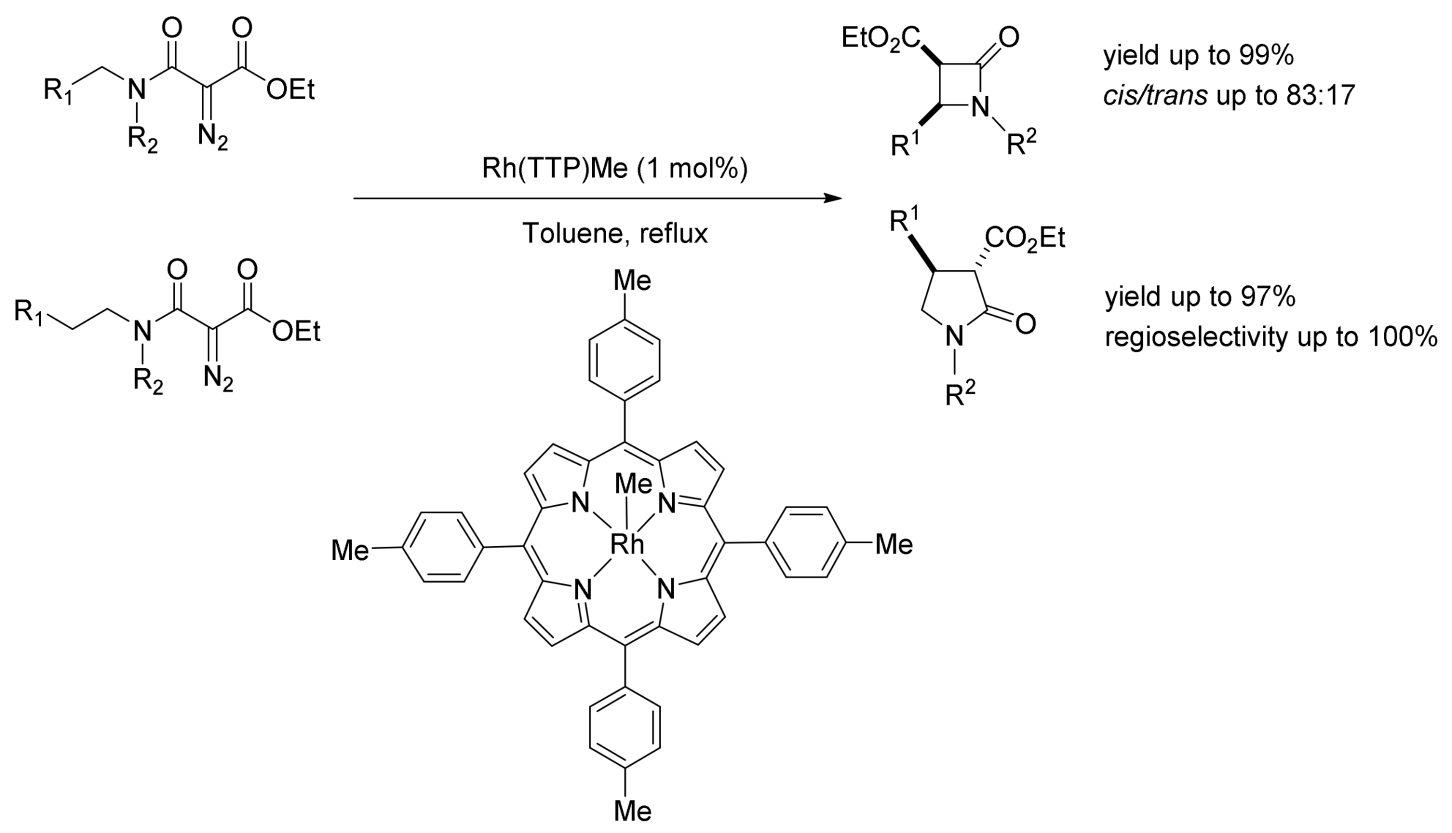
Rh(Porph)Me catalysed β-lactam and γ-lactam formation from α-diazoacetamides.

Che and co-workers also verified that different rhodium(III) porphyrin complexes are catalytically active towards stereoselective intramolecular carbene C-H insertions of α-diazoacetamides, the best catalyst being Rh(Porph)Me which gives *cis*-β-lactams or *trans*-γ-lactams in yields up to 99% with regioselectivities up to 100% and *cis/trans* ratios up to 83:17 for β-lactams (Scheme 29) [87].

**Scheme 30:**
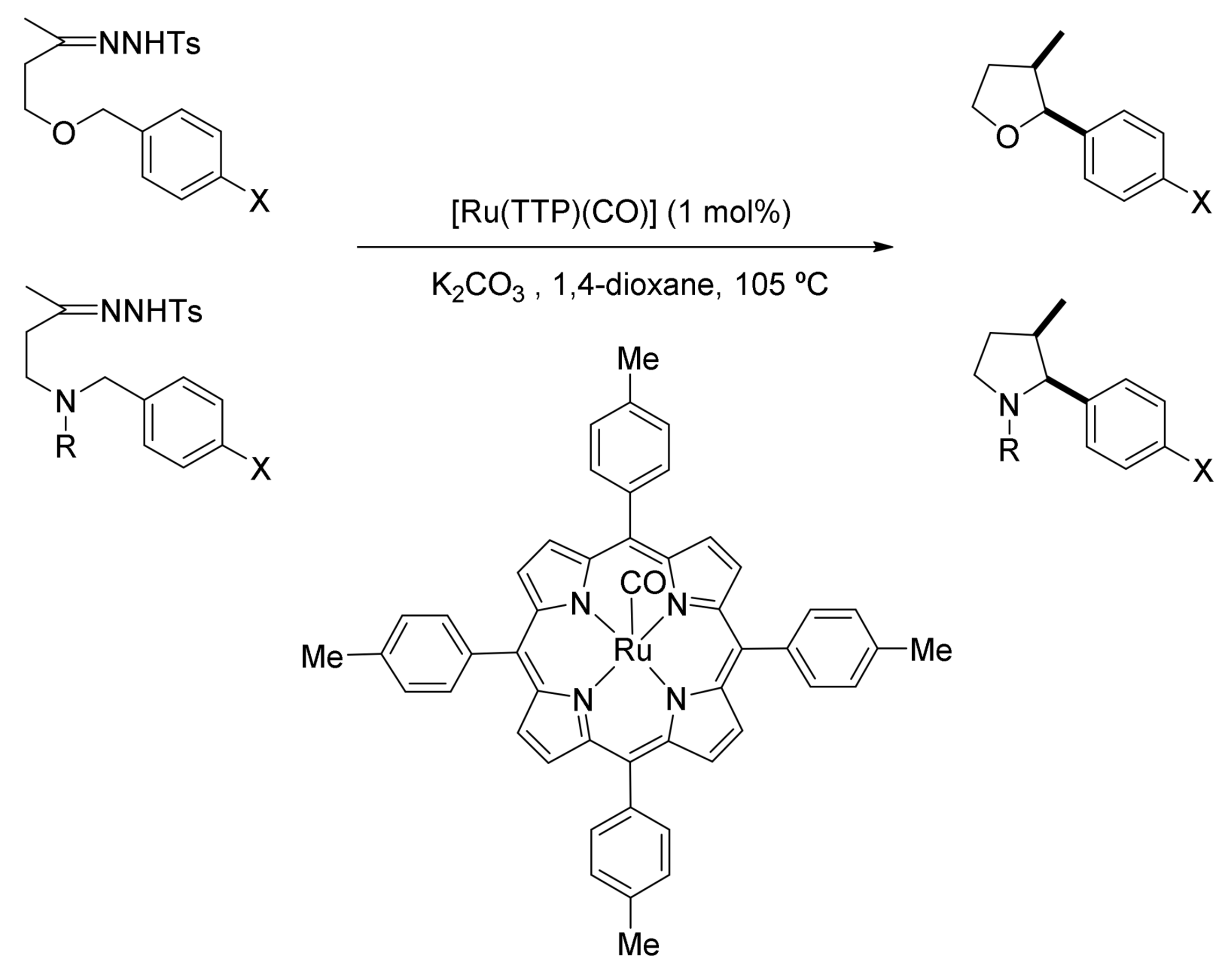
Ru(II) porphyrin catalysed cyclization of *N*-tosylhydrazones to give tetrahydrofurans and pyrrolidines.

With ruthenium porphyrin catalysts, Che and co-workers synthesized tetrahydrofurans and pyrrolidines employing alkyl diazomethanes generated in situ from *N*-tosylhydrazones through an intramolecular C(sp^3^)-H insertion of an alkyl carbene. Different catalysts and reaction conditions were tested and the best results were obtained for Ru(TTP)(CO) as catalyst, using K_2_CO_3_ as base and 1,4-dioxane as solvent. Various substituted tetrahydrofurans and pyrrolidines were synthesized in moderate to high yields (43–99%) and with moderate to excellent *cis/trans* diastereoselectivity (from 66:34 to >99:1). Moreover, the reaction worked with a variety of functionalities, including hydroxy, halo, nitro, methoxy, acetal, and alkene groups (Scheme 30) [88].

**Scheme 31:**
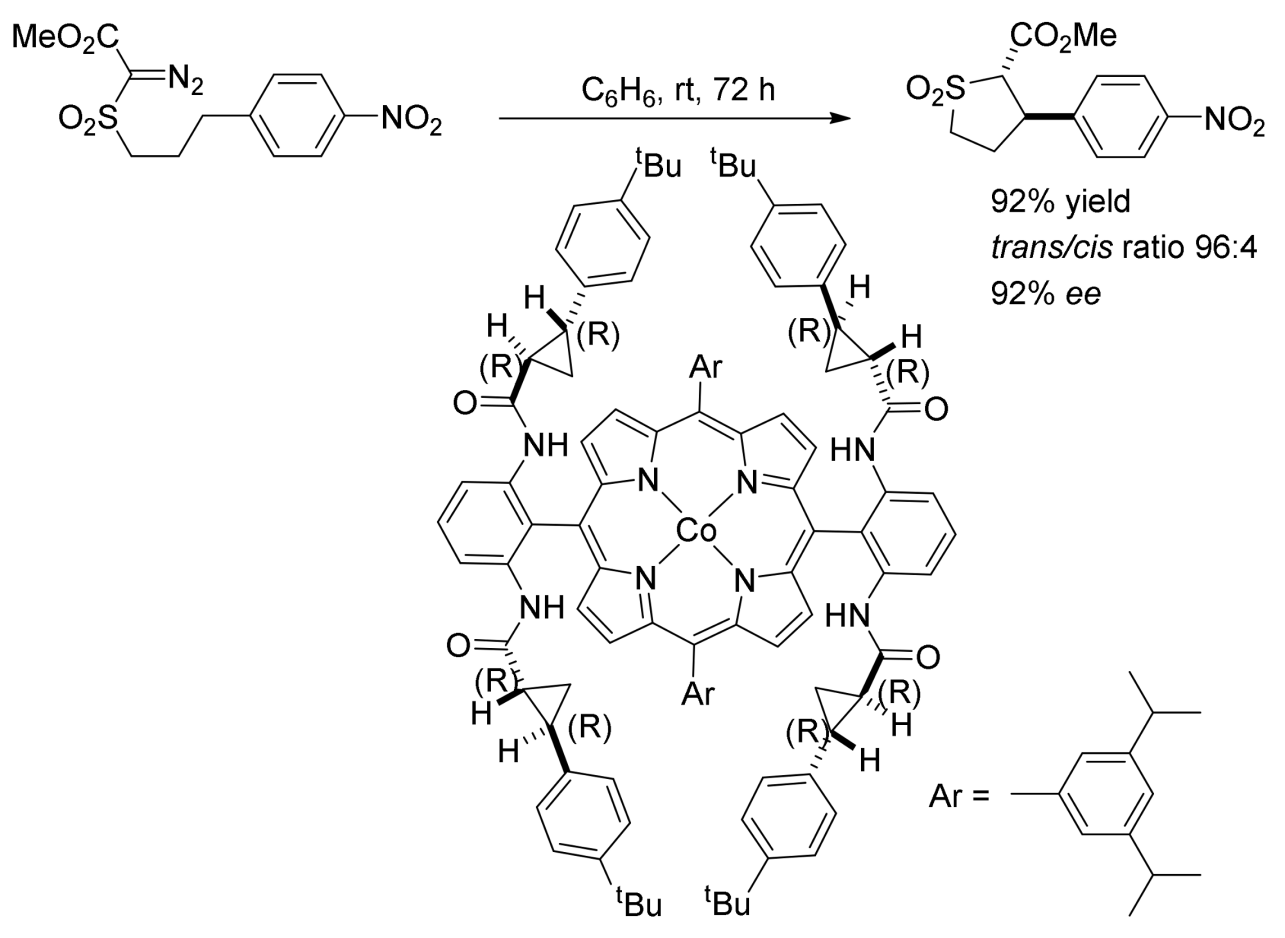
Stereoselective C-H alkylation of a α-methoxycarbonyl-α-diazosulfone compound catalysed by a chiral Co(II)-Porph.

**Figure 9:**
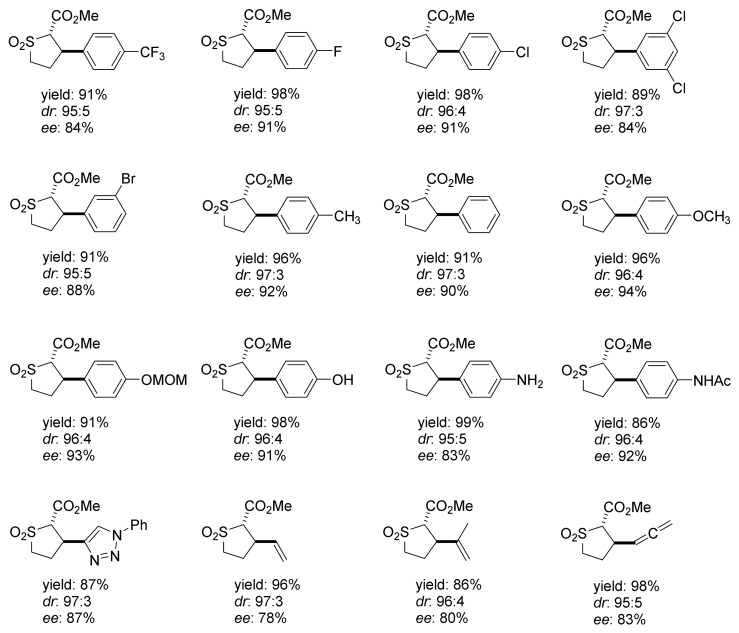
Examples of the 5-membered sulfolane derivatives resulting from the asymmetric C-H alkylation of α-methoxycarbonyl-α-diazosulfones, catalysed by a chiral Co(II) porphyrin developed by Zhang and co-workers [89].

Zhang and co-workers have designed and synthesized a D_2_-symmetric chiral amidoporphyrin as the supporting ligand for a Co(II)-based metalloradical system capable of 1,5-C-H alkylation of α-methoxycarbonyl-α-diazosulfones (acceptor/acceptor-substituted diazo reagents) with a broad range of electronic properties at ambient temperature, resulting in the 5-membered sulfolane derivatives (Scheme 31). As can be seen in Figure 9, the yields range from 86 to 99% at high diastereoselectivities (*dr* = diastereoisomeric ratio) with enantiomeric excesses (*ee*) ranging from 78 to 94%, featuring a remarkable degree of functional group tolerance [89].

## 5. Carbene Insertion into N-H

Fe(TPP)Cl was employed as catalyst for tandem N-H insertion/cyclization reactions when 1,2-diamines and 1,2-alcoholamines were treated with EDA, using dichloromethane as solvent at ambient temperature, resulting in piperazinones, morpholinones and related analogues such as quinoxalinones and benzoxazin-2-ones (Table 1). Ethylenediamine reacted rapidly with EDA in the presence of 1 mol% of catalyst at ambient temperature to give 2-piperazinone in 86% yield. On the contrary, ethanolamine reacted with one equivalent of EDA to give only the product resulting from an insertion at the amino group. Contrasting with the reaction of ethylenediamine with EDA, this product did not undergo subsequent cyclization, even after reaction times greater than 24 h and higher temperature. Other results are summarized in Table 1 [90].

As stated above, Che and co-workers demonstrated that the water-soluble porphyrin complex Ru^II^(4-Glc-TPP)(CO) (Figure 6) is an active catalyst in aqueous media exhibiting high activity an selectivity towards a number of carbenoid transfer reactions. Moreover, this Ru(II) catalyst was applied to site selectively catalyse the alkylation of the *N*-terminus of several peptides and to mediate the *N*-terminal modification of some proteins using a fluorescent-tethered diazo compound at 37 °C for 1 h and at pH 7.4 [78].

**Scheme 32:**
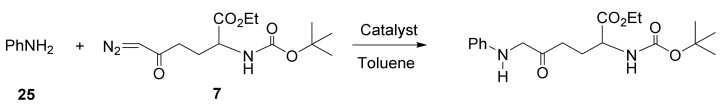
The intermolecular N-H insertion of aniline (**25**) with the *N*- and *O*-protected DON (**7**) at ambient temperature in toluene and in the presence of metalloporphyrins.

The intermolecular N-H insertion with a diazoketone such as DON was investigated by Simonneaux and co-workers with Ru(TPP)(CO) and Fe(TPP)Cl as catalysts (Figure 2). The results showed that the ruthenium complex and, to a lower extent, the iron complex are good catalysts for the transformation of aniline (**25**) into the insertion product (71% and 55% yields, respectively) (Scheme 32) [56].

**Scheme 33:**
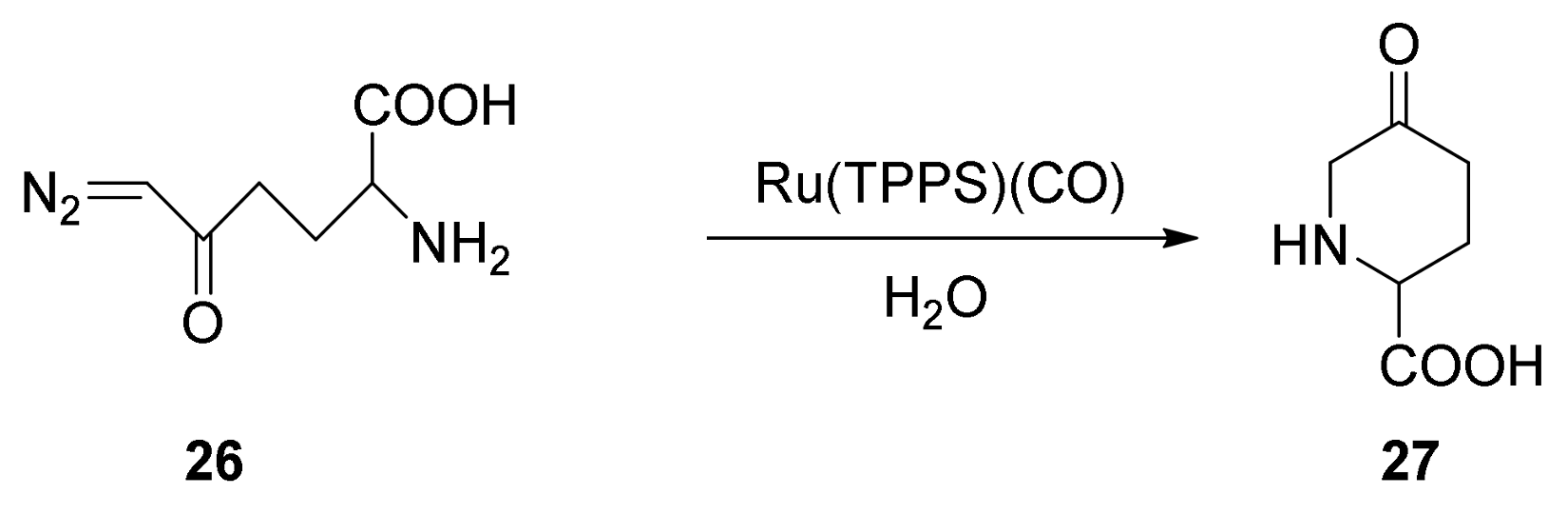
The intramolecular N-H insertion with the unprotected DON (**26**) in water at ambient temperature and in the presence of a water-soluble ruthenium porphyrin.

In the same work, Simonneaux and co-workers also described the intramolecular N-H insertion with the unprotected DON (**26**) by using a water-soluble ruthenium porphyrin as catalyst. In fact, the water-soluble Ru(TPPS)(CO) (Figure 2) in the presence of an excess of unprotected DON in water at 25 °C and under an inert atmosphere gave rise to the 5-keto-l-pipecolic acid (**27**) in 73% yield, hence resulting from an intramolecular N-H insertion in the free amino group (Scheme 33). Moreover, in the presence of 10 equivalents of styrene, and under similar reaction conditions, no cyclopropanation product was observed and only intramolecular N-H insertion was seen. Presumably as far as the two processes are concerned the intramolecular N-H insertion is much faster than the intermolecular addition of the unprotected diazo compound into the C-C double bond of styrene [56].

Most recently, Simonneaux and co-workers described the intermolecular N-H insertion of different aminoacid esters (tryptophan, phenylalanine, tyrosine) using various diazo esters (ethyl diazoacetate, diisopropyl diazomethylphosphonate, 4-(trifluoromethyl)benzyl diazoacetate and 4-(thiomethyl)benzyl diazoacetate) catalysed by Fe(TPP)Cl and by the water-soluble Ru(TPPS)(CO) and Fe(TPPS)Cl (Figure 2). Moreover, the site selective modification of terminal NH_2_ group of insulin was also accomplished by using the water-soluble Fe(TPPS)Cl as catalyst, the insertion being regioselective onto the NH_2_ termini of insulin [91].

**Scheme 34:**
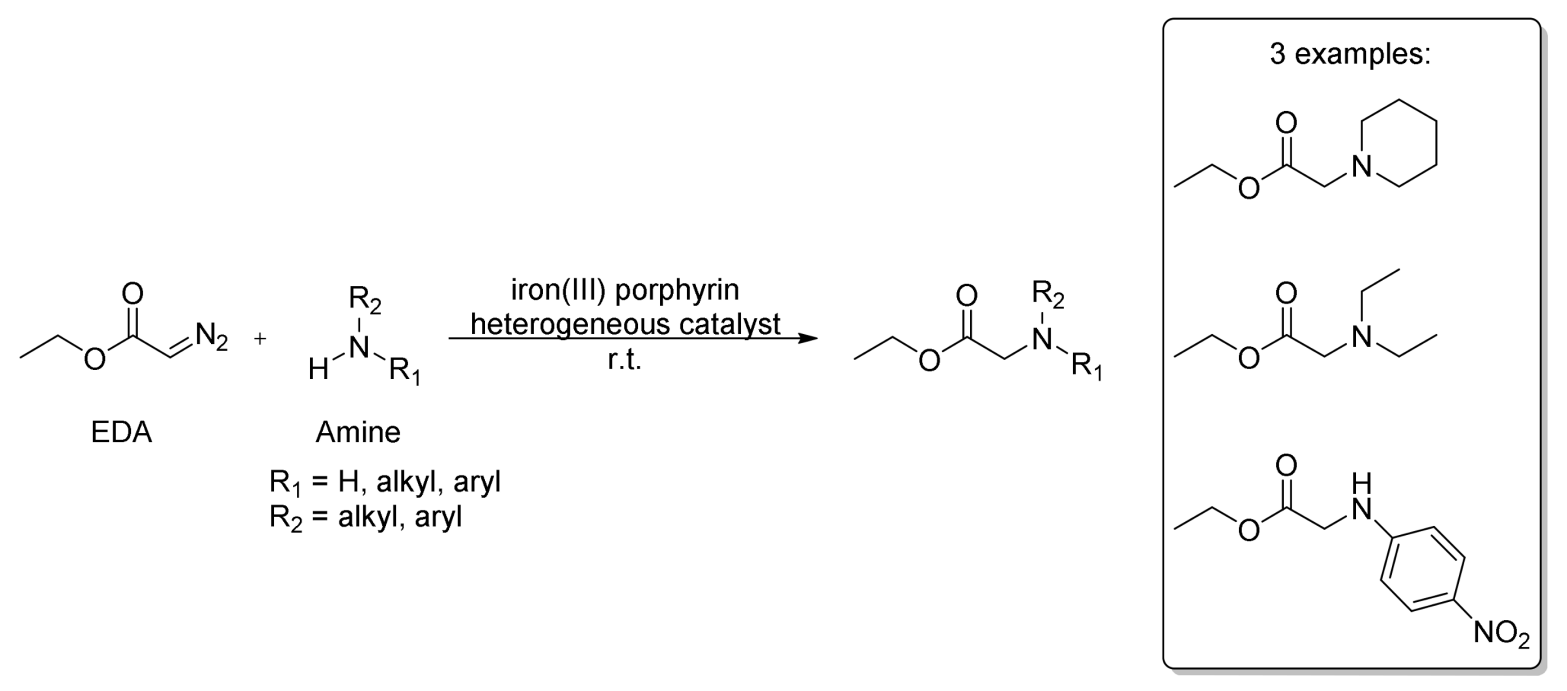
Carbene insertion into N-H bonds catalysed by iron(III) porphyrin microporous networks under heterogeneous conditions.

Son and co-workers reported the dual role of Cu_2_O nanocubes as networking catalysts and templates for hollow microporous iron porphyrin networks and their catalytic performance in carbene insertion into N-H bonds of amines. The microporous iron porphyrin networks were formed on the surface of the Cu_2_O nanocubes through an azide-alkyne cycloaddition (click reaction) of iron(III) tetrakis(4-ethynylphenyl)porphyrin chloride with 2 equivalents of 1,4-diazidobenzene. After acidic treatment, hollow microporous iron porphyrin networks were obtained, which showed excellent catalytic activity in carbene insertion into N-H bonds with EDA, maintaining its activity during five cycles. The molar ratio between the single N-H insertion product and the double N-H insertion product for primary amines was as high as 100:0 for 4-nitroaniline, whereas for 4-methoxyaniline, 4-methylaniline, aniline, 4-bromoaniline, and 4-cyanoaniline this ratio was 81:19, 90:10, 93:7, 98:2, and 99:1, respectively (Scheme 34) [92].

Earlier, the same group had already reported the catalytic carbene insertion into the N-H bonds of amines using iron(III) porphyrin microporous networks on iron oxide nanoparticles (Fe_3_O_4_) as catalyst, which can be considered as microporous organic networks. By the Sonogashira coupling of the Fe(III) tetrakis(4-ethynylphenyl)porphyrin and 1,4-diiodobenzene, the Fe_3_O_4_ nanoparticles were coated with iron(III) porphyrin networks. The resulting catalyst was easily recycled from the reaction mixture by magnetic separation, giving 100% yield for piperidine in four consecutive runs, affording only the single N-H insertion product (Scheme 34) [93].

Using another methodology, Huang and co-workers have prepared Fe(TPP)Cl functionalized microporous organic nanotube networks that were employed as heterogeneous catalysts for carbene insertion into N-H bonds with EDA and presented high catalytic activity and reusability. The heterogeneous catalyst was used for 12 cycles almost without loss of activity in the catalytic N-H insertion of 4-bromoaniline with EDA. The N-H insertion reaction also presented the formation of mono and double N-H insertion products, with high selectivity for mono insertion (Scheme 34) [94].

Azidopropyl functionalized mesoporous silicas SBA-15 were prepared with variable azide loadings and these materials were functionalized selectively with ethynylated organic moieties through a copper-catalysed azide alkyne cycloaddition or click reaction. One of those immobilizations involved the iron(III) porphyrin (FeTPP)Cl via click reaction with ethynyl-H_2_TPP. Heterogeneous iron(III) porphyrin catalysed carbene insertion into a N-H bond was studied using piperidine and ethyl diazoacetate (EDA) as benchmark reaction, affording high yields after 2 h of reaction [95]. The N-H insertion procedures applied to amines were used for the synthesis of amino acids. 

Ir(TTP)CH_3_ also catalyses the N-H insertion reactions between EDA or MPDA and aryl, aliphatic, primary, and secondary amines. The products are substituted glycine esters. With arylamines obtained yields were above 80% or higher but for reaching such values it was necessary to avoid the slow addition of the diazo derivative. Aliphatic amines also provided good product yields, still in some cases higher catalyst loadings and slow amine addition were required. Both single and double insertion compounds were obtained from primary amines and EDA; however, by choosing the adequate stoichiometric ratio and reaction temperature, each single or double insertion product could be selectively obtained. The insertion of 1 equiv. of EDA and 1 equiv. of MPDA into primary amines gave rise to trisubstituted amines like RN(CH_2_CO_2_Et)(CHPhCO_2_Me) were produced. According to Woo and co-workers, the Ir(TTP)CH_3_ catalyses N-H insertion reactions; mechanistic studies support a stepwise mechanism involving a metal-ylide intermediate, formed by the nucleophilic attack of the amine onto the intermediate carbene complex [96,97]. According to Shaik and co-workers, also the iron(III) porphyrin carbene’s N-H insertion reactions follow a nucleophilic attack pathway, forming an ylide as intermediate [98].

**Scheme 35:**
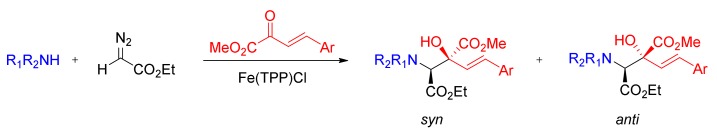
Iron(III) porphyrin catalysed three-component reaction.

Furthermore, Hu and co-workers were able to trap this metal-ylide intermediate with suitable electrophiles for developing a three-component reaction, thus providing further evidence for the existence of an ylide intermediate in iron porphyrin catalysed N-H insertion processes, along with the synthesis of β-hydroxy-α-amino esters starting from ethyl diazoacetate and aliphatic amines (Scheme 35) [99].

## 6. Carbene Insertion into O-H

**Scheme 36:**
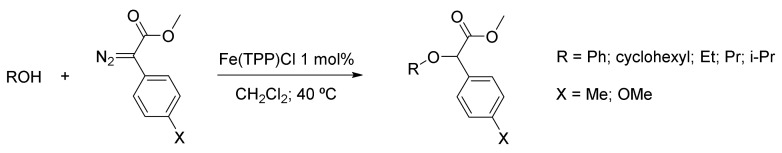
Insertion of carbenes derived from methyl 2-phenyldiazoacetates into O-H bonds catalysed by Fe(TPP)Cl.

Fe(TPP)Cl efficiently catalysed the insertion of carbenes derived from methyl 2-phenyldiazoacetates (MPDAs) into O-H bonds of aliphatic and aromatic alcohols, affording products in good yields such as 56% for propan-2-ol, 64% for ethanol, 83% for phenol, and 88% for cyclohexanol. Nevertheless, when EDA was tested as the carbene source for O-H insertion reactions using Fe(TPP)Cl as catalyst, only maleates and fumarates were formed. This is in contrast to equivalent N-H insertions where EDA showed to be an excellent carbene source. The O-H insertion reactions required heating in refluxing CH_2_Cl_2_ for about 8 h using 1 mol% of the Fe(TPP)Cl catalyst (Scheme 36) [90].

**Scheme 37:**
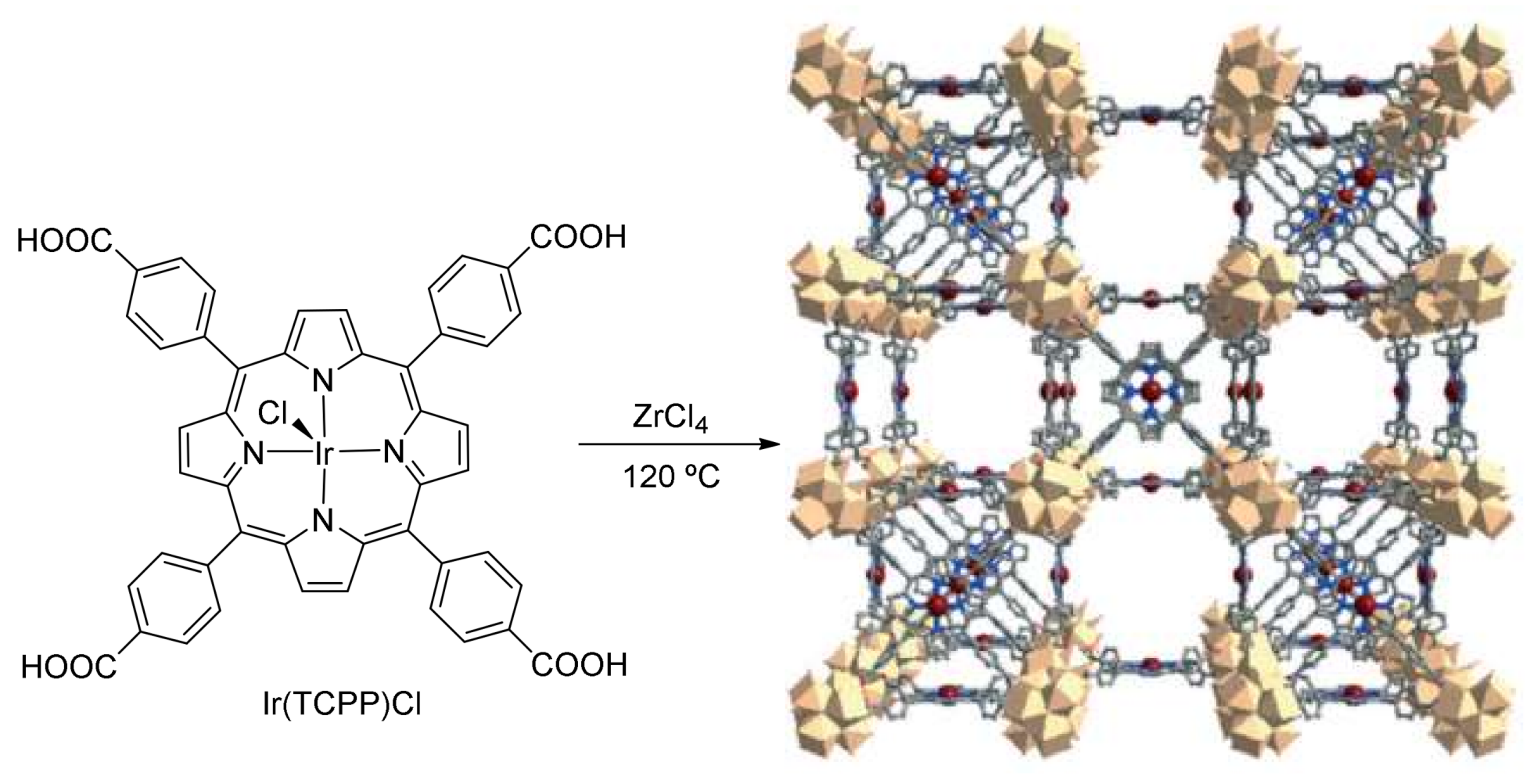
IrPMOF(Zr) synthesis by self-assembly of Ir(TCPP)Cl with ZrCl_4_. Adapted from Ref. [100] with permission of The Royal Society of Chemistry.

An iridium(III) porphyrin was embedded into a porous zirconium metal-organic framework [IrPMOF(Zr)] that was synthesized by self-assembly of an iridium(III) porphyrin tetracarboxylic ligand Ir(TCPP)Cl [TCPP = tetrakis(4-carboxyphenyl)porphyrin] with ZrCl_4_ in the presence of benzoic acid (Scheme 37). This heterogeneous catalyst was used to promote carbene transfer into O-H bonds of alcohols and phenols with EDA at ambient temperature. The product yields obtained after 10 min of reaction range from 56% for phenol to 94% for propan-2-ol, with a turnover frequency (TOF) up to 4260 h^-1^ determined for propan-2-ol. This IrPMOF(Zr) catalyst could be recycled and reused for 10 cycles without significant loss of catalytic activity [100].

## 7. Carbene Insertion into S-H

**Scheme 38:**
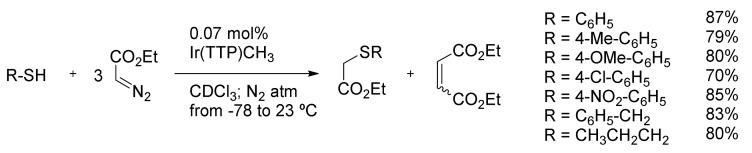
Reaction of EDA with aromatic and aliphatic thiols catalysed by Ir(TTP)CH_3_ [101].

The insertion of carbenes derived from diazo esters into the S-H bonds of aromatic and aliphatic thiols was catalysed by (5,10,15,20-tetratolylporphyrinato)methyliridium(III), Ir(TTP)CH_3_, at low reaction temperatures. Ethyl diazoacetate (EDA), methyl diazoacetate (MDA), methyl phenyldiazoacetate (MPDA), and methyl (*p*-tolyl)-diazoacetate (MTDA) were used and the yields of the resulting thioethers were as high as 97% for aromatic thiols, with catalyst loadings as low as 0.07 mol%. When a CDCl_3_ solution of benzenethiol was treated with EDA in the presence of a catalytic amount of Ir(TTP)CH_3_, the carbene fragment from EDA readily inserted into the S-H bond to generate ethyl 2-(phenylthio)acetate in 87% yield within 15 min. The only observed by-products were diethyl maleate and diethyl fumarate, resulting from carbene dimerization (Scheme 38) [101].

**Scheme 39:**
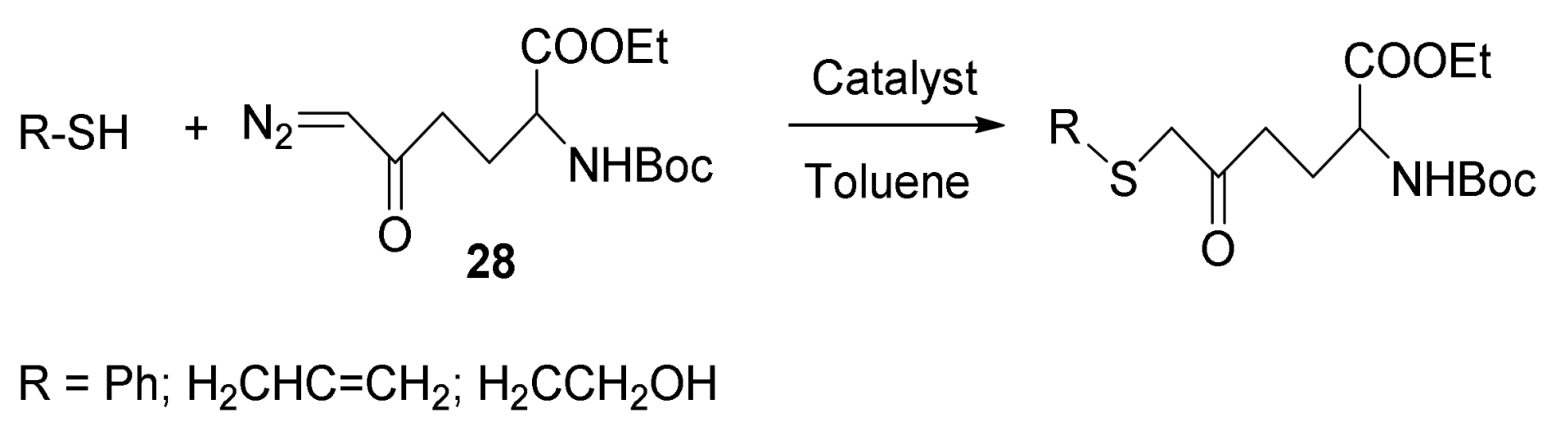
Reaction of the protected DON (**28**) with thiols catalysed by Ru(TPP)(CO) or Fe(TPP)Cl.

The treatment of thiophenol with *N*-*tert*-butoxycarbonyl-6-diazo-5-oxo-l-norleucine (DON) ethyl ester (**28**), catalysed by Ru(TPP)(CO) and Fe(TPP)Cl, gave insertion of the diazo derivative into the S-H bond with 85% and 51% yield, respectively (Scheme 39). To fully evaluate the reactivity of this diazoketone, a study involving the competition between the cyclopropanation of olefins and the insertion reaction catalysed by Ru(TPP)(CO) or Fe(TPP)Cl, was also undertaken. Only the S-H insertion compound was observed when allyl thiol was the substrate with 87% and 58% yield, respectively. Also in order to completely understand the catalytic abilities of Ru(TPP)(CO) or Fe(TPP)Cl, another study about the possible competition between the S-H and O-H insertions was also undertaken. Only S-H insertion took place for 2-mercaptoethanol [56].

## 8. Carbene Insertion into Si-H

Che and co-workers observed also that the chiral metalloporphyrin Ir(Halt)Me(L) (Figure 7) is able to enantioselective Si-H bond insertion with Et_3_SiH at −80 °C for 24 h in CH_2_Cl_2_, affording product yields from 75 to 91% and 75–91% *ee*. The authors also reported that the insertion of α-aryl-α-diazoesters into PhMe_2_SiH in the presence of the Ir(III) porphyrin complex Ir(Halt)Me(L) proceeded with good yields (75–92%) and enantioselectivities (72–91%) [83].

The iridium(III) porphyrin Ir(TCPP)Cl was embedded into a porous hafnium metal-organic framework [IrPMOF(Hf)] that was synthesized by reacting the iridium(III) porphyrin tetracarboxylic ligand with HfCl_4_. The porous [IrPMOF(Hf)] can promote the selective carbenoid insertion reactions into Si-H bonds with EDA under heterogeneous conditions. An inverted reactivity and selectivity order of Si-H insertion reactions (primary > secondary > tertiary), which is unattainable by conventional metal catalysts, is achieved, and the MOF catalyst is easily separated and recycled, with the yield after 10 runs being similar to that of the first run [102].

## 9. Alkene Formation Reactions

### 9.1. Coupling of Diazo Compounds

**Scheme 40:**
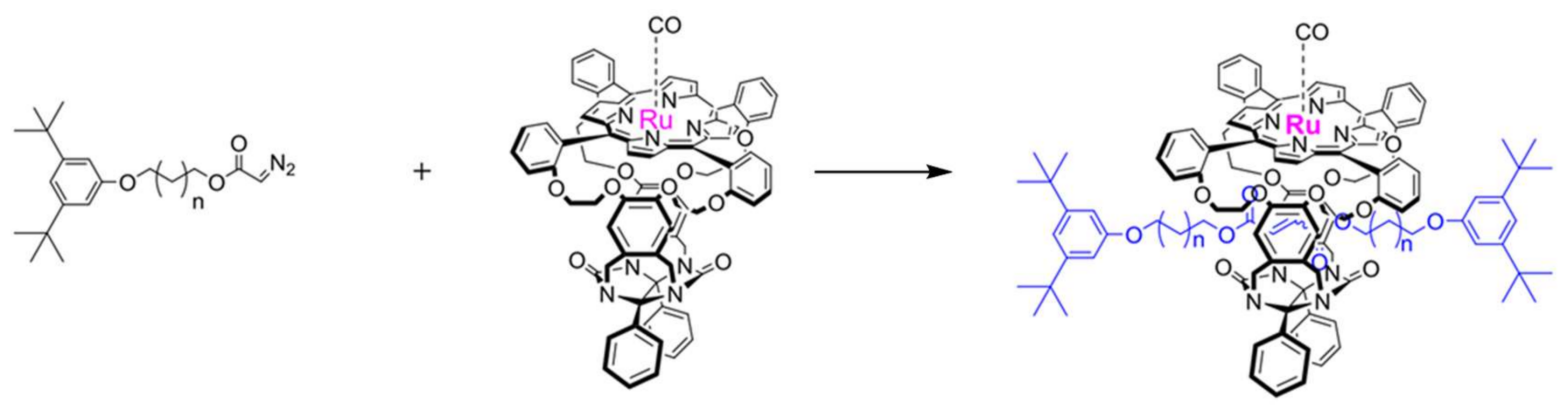
Synthesis of rotaxanes from α-diazoesters. Adapted from reference [103] with permission of Elsevier.

Nolte and co-workers [103] have demonstrated that a ruthenium porphyrin functionalized with a cavity based on diphenylglycoluril can serve as a carbene transfer reaction catalyst for the dimerization of α-diazoesters inside its cavity. When these α-diazoesters contain bulky blocking groups, rotaxanes are formed (Scheme 40).

A short alkyl spacer (C-3) between the α-diazoester moiety and the blocking group yields a rotaxane with a linear thread composed of two carbene moieties. When a longer spacer (C-6) is used, additional reactions of the α-diazoester with the product rotaxane give higher molecular mass products, in which up to 4 extra carbenes are incorporated into the initially formed rotaxane thread. NMR and MS studies indicated that these reactions involved C=C and C-H insertions. The fact that the rotaxane with short C-3 alkyl spacers is inert towards such insertion reactions is attributed to the restricted access of additional α-diazoesters to its cavity, due to the close proximity of the bulky 3,5-di-*tert*-butylphenyl blocking groups [103].

### 9.2. Olefination of Carbonyl Compounds

**Scheme 41:**
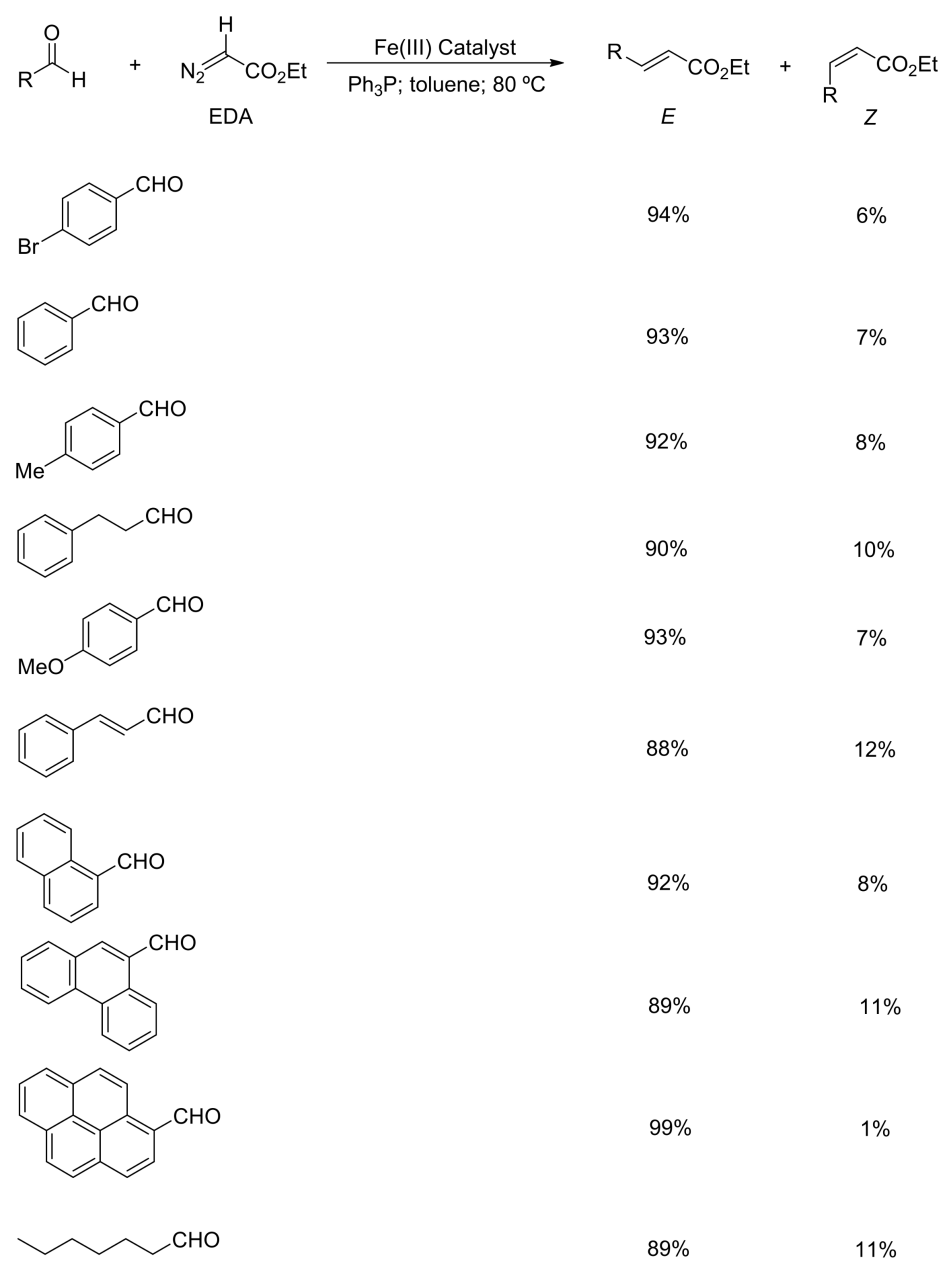
Heterogeneous catalytic olefination of aldehydes catalysed by Fe(TPP)Cl microporous organic nanotube networks.

As referred above, Huang and co-workers [94] were able to prepare microporous organic nanotube networks incorporating Fe(TPP)Cl, materials that were employed as heterogeneous catalysts also in the olefination of aldehydes with EDA, displaying good catalytic performance and reusability (up to 7 cycles for 4-bromobenzaldehyde, decreasing to 71% in the last run), since nearly no iron leaching occurred during the course of the reactions. The presence of electron-withdrawing groups increases the reactivity with shorter reaction times (Scheme 41).

On the contrary, the less reactive electron-rich benzaldehydes could be quantitatively olefinated but in longer reaction times. For example, 4-methoxybenzaldehyde was the least reactive and the reaction could be completed only after a prolonged reaction time (6 h). This Fe(TPP)Cl heterogeneous catalyst is also suitable for non-aromatic aldehydes such as α,β-unsaturated and aliphatic aldehydes. Some aromatic aldehydes with their various molecular sizes were also studied; the results have shown that when the molecular size rises, the reaction times are longer and the yields of olefination are lower. This was attributed to a possible obstruction resulting from the bigger molecular sizes that hamper molecules’ diffusion and accessibility to the active sites of the catalysts’ microporous organic nanotube network (Scheme 41) [94].

## 10. Conclusions

The present review highlights the most recent developments concerning synthetic metalloporphyrin-catalysed carbene transfer reactions and their significance in organic synthesis. The biomimetic catalytic activity demonstrated by synthetic metalloporphyrins was recognized decades ago for several important transformations, and carbene transfer reactions represent one of the most fascinating fields where metalloporphyrins are active as catalysts. Accordingly, this review encompasses key organic transformation reactions catalysed by metalloporphyrins, such as cyclopropanation, cyclopropenation, carbene C-H, N-H, O-H, S-H, and Si-H insertion, ending with olefination of carbonyl compounds. All topics were exhaustively explored in the literature since the year 2010.

## Figures and Tables

**Table 1:** Results obtained in tandem N-H insertion/cyclization reactions of 1,2-disubstituted substrates with EDA catalysed by Fe(TPP)Cl in CH_2_Cl_2_.

1,2-Disubstituted Substrates	EDA (equiv.)	Product (s)
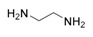	1	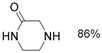
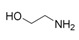	1	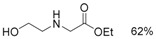
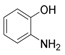	1	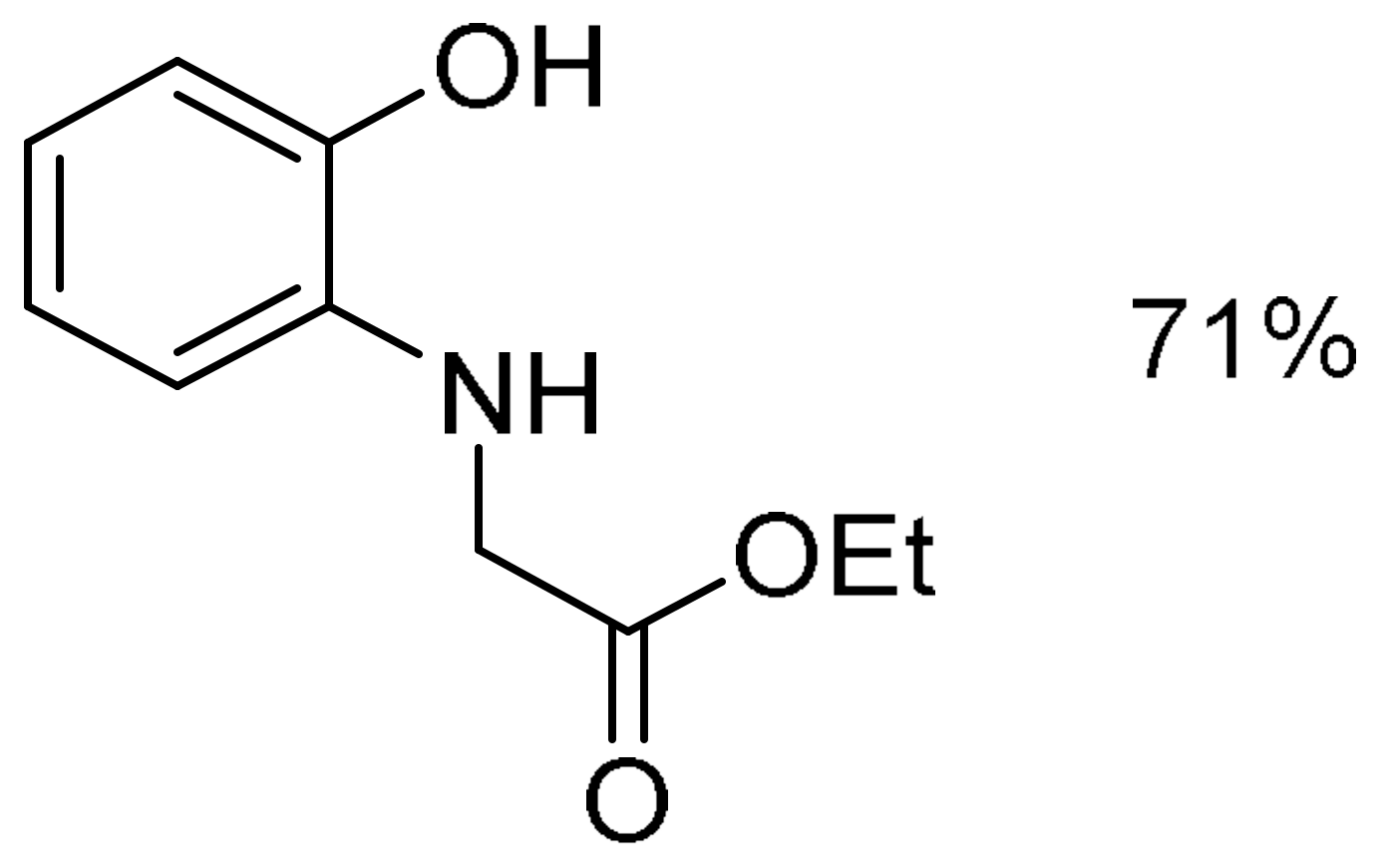
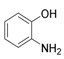	2	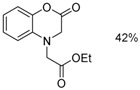
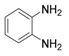	1	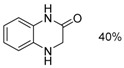
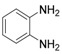	2	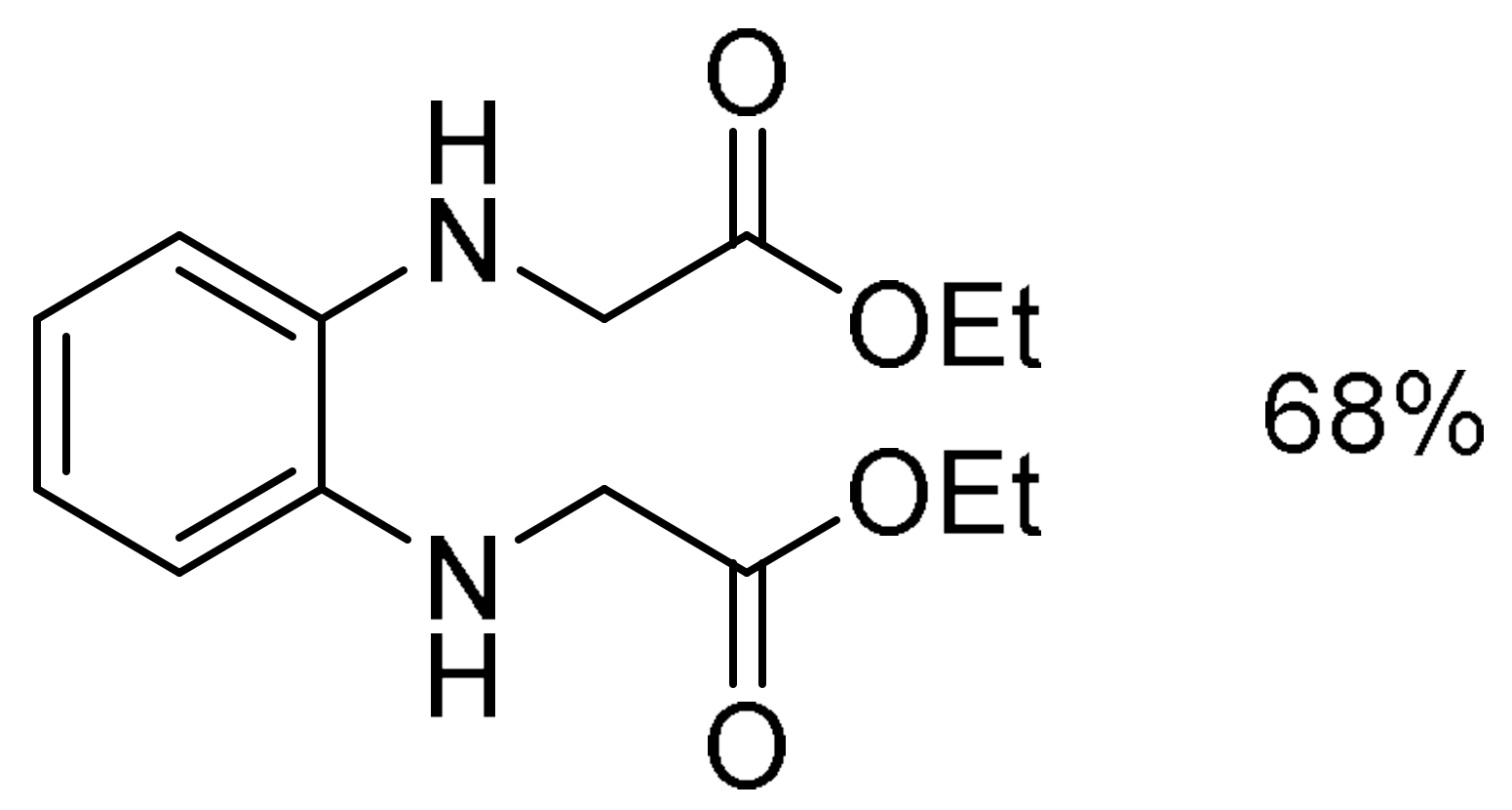
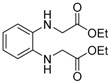	60 ° C12 h (without EDA)	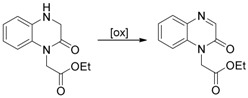
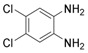	2